# Lack of prion transmission barrier in human PrP transgenic *Drosophila*

**DOI:** 10.1016/j.jbc.2024.107617

**Published:** 2024-07-30

**Authors:** Alana M. Thackray, Erin E. McNulty, Amy V. Nalls, Andrew Smith, Emmanuel Comoy, Glenn Telling, Sylvie L. Benestad, Olivier Andréoletti, Candace K. Mathiason, Raymond Bujdoso

**Affiliations:** 1 Department of Veterinary Medicine, University of Cambridge, Cambridge, UK; 2 Prion Research Center (PRC) and the Department of Microbiology, Immunology and Pathology, Colorado State University, Fort Collins, Colorado, USA; 3 Commissariat à l'Energie Atomique, DRF/IBFJ/SEPIA, Fontenay-aux-Roses, France; 4 WOAH Reference Laboratory for CWD (SLB), Department of Biohazard and Pathology, Norwegian Veterinary Institute, Ås, Norway; 5 UMR INRA ENVT 1225 -Hôtes-Agents Pathogènes, Ecole Nationale Vétérinaire de Toulouse, Toulouse, France

**Keywords:** BSE, chronic wasting disease, *Drosophila*, human, neurodegenerative disease, nonhuman primate, prion, transgenic, vCJD, zoonotic

## Abstract

While animal prion diseases are a threat to human health, their zoonotic potential is generally inefficient because of interspecies prion transmission barriers. New animal models are required to provide an understanding of these prion transmission barriers and to assess the zoonotic potential of animal prion diseases. To address this goal, we generated *Drosophila* transgenic for human or nonhuman primate prion protein (PrP) and determined their susceptibility to known pathogenic prion diseases, namely varient Creutzfeldt–Jakob disease (vCJD) and classical bovine spongiform encephalopathy (BSE), and that with unknown pathogenic potential, namely chronic wasting disease (CWD). Adult *Drosophila* transgenic for M129 or V129 human PrP or nonhuman primate PrP developed a neurotoxic phenotype and showed an accelerated loss of survival after exposure to vCJD, classical BSE, or CWD prions at the larval stage. vCJD prion strain identity was retained after passage in both M129 and V129 human PrP *Drosophila*. All of the primate PrP fly lines accumulated prion seeding activity and concomitantly developed a neurotoxic phenotype, generally including accelerated loss of survival, after exposure to CWD prions derived from different cervid species, including North American white-tailed deer and muntjac, and European reindeer and moose. These novel studies show that primate PrP transgenic *Drosophila* lack known prion transmission barriers since, in mammalian hosts, V129 human PrP is associated with severe resistance to classical BSE prions, while both human and cynomolgus macaque PrP are associated with resistance to CWD prions. Significantly, our data suggest that interspecies differences in the amino acid sequence of PrP may not be a principal determinant of the prion transmission barrier.

Prion diseases, or transmissible spongiform encephalopathies, are fatal neurodegenerative conditions that affect a range of mammalian species. These conditions include human prion diseases such as kuru, Creutzfeldt–Jakob disease (CJD), and Gerstmann–Straussler–Scheinker syndrome (GSS), bovine spongiform encephalopathy (BSE), scrapie of sheep, and chronic wasting disease (CWD) of cervids (1). These diseases appear to share the same pathogenic mechanism, which involves the misfolding and aggregation of the normal host prion protein normal cellular PrP (PrPC) into abnormal disease-specific conformation of PrP (PrPSc) (1). Misfolded prion protein (PrP) accumulates principally in the central nervous system of affected individuals where spongiform-like vacuolation, neuronal loss, reactive proliferation of astroglia, and PrPSc deposition are commonly seen. The protein only (prion) hypothesis has proposed that the moiety responsible for transmission of these conditions is composed principally of PrPSc, lacks a nucleic acid and is referred to as a prion (2). Strong support for this hypothesis has been provided by the demonstration that prions can be generated from recombinant PrP *in vitro* that are infectious *in vivo* (3, 4, 5, 6, 7, 8).

The transmissible nature of prion diseases was established by experimental passage of scrapie in sheep (9) and goats (10), and by transmission of kuru and CJD in chimpanzees (11, 12). Further studies, particularly in rodents, showed that interspecies primary passage prion transmissions generally show inefficient attack rates and prolonged incubation times, or even subclinical disease. This was attributed to a prion transmission barrier effect (13, 14, 15, 16), a phenomenon believed to be regulated principally by interspecies PrP amino acid sequence differences (17, 18) and influenced by the prion strain attempting transmission (19, 20). The conformational selection model proposes that a particular PrPC amino acid can support propagation of only a limited subset of PrPSc conformations (21). In this context, prion propagation is believed to be governed by the overlap of donor and host PrP conformations, and only where this occurs does efficient disease transmission result. Animal prion diseases were considered a zoonotic threat to public health through the human food chain. However, lack of reproducibility of experimental transmission of classical sheep scrapie to nonhuman primates, including chimpanzee (22, 23, 24), and the absence of epidemiological data to link scrapie to human prion disease (25, 26) was regarded as evidence of a sufficiently robust transmission barrier that prevented animal prion diseases being zoonoses. This view has since changed with the realisation that variant CJD (vCJD) in humans arose because of the BSE epizootic in cattle (27, 28, 29, 30). Human PrP is polymorphic at codon 129 where either methionine (Met_129_) or valine (Val_129_) can be encoded. To date, all but one clinical and neuropathologically confirmed vCJD cases have been Met_129_ homozygous. This implied that Val129 human PrP provides a significant transmission barrier to the propagation of BSE prions, which has been confirmed by bioassay in human PrP transgenic mice. The discovery of a new animal prion disease in camels (31) and the identification of CWD in Northern Europe (32, 33) has renewed attention on assessment of the zoonotic potential of animal prion diseases (34). This is important in order to identify those animal prion diseases that are pathogenic and those that are innocuous to human health.

CWD is a prion disease of free-ranging and captive cervid species including deer, elk, moose, and reindeer (35). First identified in Colorado and Wyoming in the USA in the 1960s, CWD has since been identified in 33 states of the USA and five Canadian provinces (https://www.usgs.gov/centers/nwhc/science/expanding-distribution-chronic-wasting-disease). North American CWD is highly contagious since secretion and excretion of prion infectivity from affected cervids is believed to result in environmental contamination and subsequent efficient lateral transmission (36). A small number of cases of imported CWD have been identified in captive cervids in South Korea following the import of deer from Canada (37). Since 2016, CWD was identified collectively in Nordic free-ranging cervids (moose, red deer, reindeer) in Norway and in moose in Finland and Sweden (32, 33). This geographical expansion of CWD occurrence, coupled with its potential for contagious spread in cervids, has raised issues about transmission of the disease to other species, including humans. It has been shown that CWD isolates can be successfully experimentally transmitted to farmed animals (38, 39, 40). However, the apparent failure of cervid prions to propagate in cynomolgus macaques and human PrP transgenic mice has suggested that an effective transmission barrier restricts the zoonotic potential of CWD in humans (34, 41).

The use of mammalian experimental hosts to model the human transmission barriers for animal prions is cumbersome, time consuming, expensive and, particularly in the case of nonhuman primates, subject to increasing ethical debate. Consequently, it is important to develop new animal systems that can be used to contribute to the assessment of the zoonotic potential of animal prion diseases such as CWD. To address this issue, we have developed *Drosophila melanogaster* as a new tractable animal model to study mammalian prion biology. We have previously shown that prion-exposed PrP transgenic *Drosophila* display core features of mammalian prion disease, namely progressive accumulation of transmissible prion infectivity accompanied by the increasing severity of a neurotoxic phenotype, evident as impaired locomotor ability and accelerated loss of survival (42, 43, 44). In addition, the products of protein misfolding cyclic amplification (PMCA) reactions seeded with mammalian prion-exposed PrP *Drosophila* head homogenate displayed proteinase K-resistant PrP 27–30 (42). These studies have validated PrP transgenic *Drosophila* as a new invertebrate host to study the biology of transmissible mammalian prion disease.

The aim of our study presented here was 2-fold. Firstly, to investigate whether PrP transgenic *Drosophila* displayed similar transmission barriers for animal prion propagation that are observed in mammalian hosts. Secondly, to determine whether PrP transgenic *Drosophila* can contribute to assessment of the zoonotic potential of cervid CWD. To do so, we generated *Drosophila* transgenic for either M129 or V129 human PrP, or chimpanzee, cynomolgus macaque, squirrel monkey, or lemur PrP expressed pan neuronally in the fly. We found that M129 and V129 human PrP transgenic *Drosophila* were equally susceptible to classical BSE and to vCJD prion infection, and that the identity of the vCJD prion strain was retained after passage in both fly lines. In addition, human and nonhuman primate PrP transgenic *Drosophila*, including those that expressed cynomolgus macaque PrP, were susceptible to cervid CWD prions after exposure to North American and European CWD isolates. These novel studies show that *Drosophila* transgenic for human or nonhuman primate PrP lack functional prion transmission barriers that are observed when the same PrP amino acid sequence is expressed in the natural host. Furthermore, our data suggest that interspecies differences in amino acid sequence of PrP are not the principal determinant of the cross-species prion transmission barrier. Collectively, our data highlight an important role for PrP transgenic *Drosophila* as a new animal model for use in the study of biology of the prion transmission barrier.

## Results

**Figure 1:**
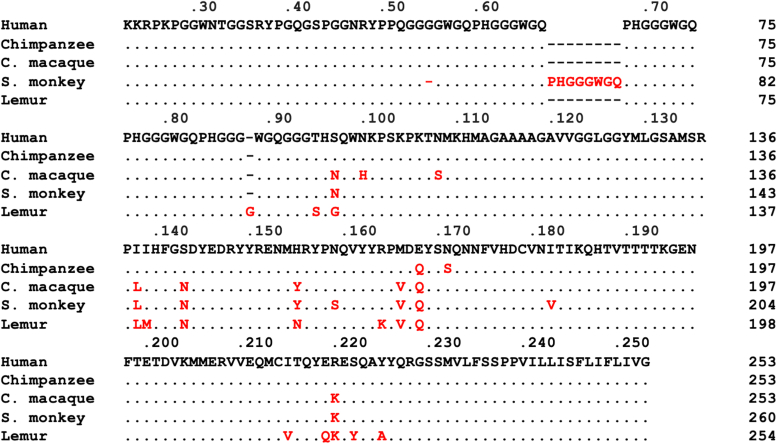
**Amino acid sequence comparison of primate PrP.** Amino acid sequence alignment of human (M129) and nonhuman primate PrP sequences. The nonhuman primate species used in the comparison were chimpanzee, cynomolgus macaque (C. macaque), squirrel monkey (S. monkey), and lemur. The PrP sequence for each species corresponds to the amino acid sequences for the mature PrP and the subsequent GPI anchor signal sequence. The nonhuman primate PrP amino acid sequences were compared to that of human PrP. Consensus residues are represented by a *dot* (.), while nonconsensus residues are indicated by the corresponding amino acid colored in *red*. The numbers above the sequence lines correspond to the amino acid numbering for the full-length human PrP, while the numbers to the right of each sequence line correspond to the amino acid numbering for the full-length PrP of the corresponding species. GPI, glycosylphosphatidylinositol; PrP, prion protein.

We have investigated the use of *Drosophila* transgenic for human or nonhuman primate PrP in order to model the zoonotic potential of cervid CWD prions. The panel of primate PrP *Drosophila* used here included M129 or V129 human PrP *Drosophila*, or *Drosophila* transgenic for chimpanzee, cynomolgus macaque, squirrel monkey, or lemur PrP. The data in Figure 1 show the amino acid sequence alignment of the human (M129) and nonhuman primate PrP sequences used here. Overall, the primate PrP amino acid sequences show >92% similarity and possess characteristic features described for other mammalian species forms of PrP including multiple octapeptide repeats in the N-terminal region and three predicted alpha-helices in the C-terminal region, with helix-1 flanked by short beta-strand regions. Compared to the human PrP amino acid sequence, chimpanzee PrP contained two substitutions, cynomolgus macaque contained nine substitutions, squirrel monkey PrP had one deletion, insertion of an octapeptide repeat region and contained nine substitutions, while lemur PrP contained an insertion of one additional amino acid residue and 14 substitutions. The amino acid substitutions in nonhuman primate PrP were generally located in the C-terminal portion of the protein in the region of helix-2 and helix-3 but not helix-1. All the nonhuman primate PrP amino acid sequences investigated here contained a methionine residue at the equivalent position to codon 129 in human PrP. The phylogenetic relatedness of nonhuman primate PrP to human PrP was chimpanzee > cynomolgus macaque > squirrel monkey > lemur based on the above analysis and that by others (45).

**Figure 2:**
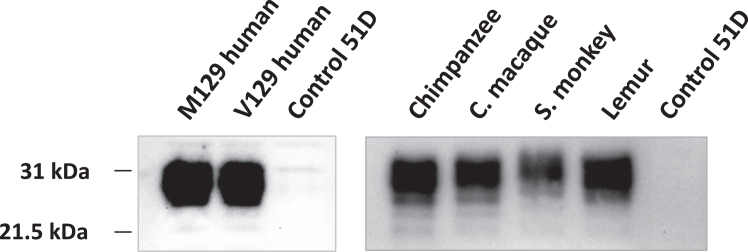
**Western blot detection of prion protein expression in primate PrP *Drosophila.****Elav* x primate PrP or *Elav* x control 51D *Drosophila* were harvested at 5 days of age. Fly head homogenates were prepared and analyzed by SDS-PAGE and Western blot with anti-PrP mAb Sha31 as described in the Experimental procedures. Molecular mass marker values (kDa) are shown on the *left-hand side*.

We generated transgenes that encoded human or nonhuman primate PrP amino acid sequences shown in Figure 1 that were subsequently inserted into the *Drosophila* genome. PrP transgenesis was mediated by pUASTattB/PhiC31 site–specific transformation, which leads to insertion of a single copy of each transgene at the same genomic landing site in each of the different fly lines (46, 47). The Western blot data in Figure 2 show that the different genotypes of primate PrP were efficiently expressed in *Drosophila* and at a similar level in each fly line with a molecular profile equivalent to that seen for other mammalian forms of PrP expressed in *Drosophila* (44, 48, 49). The molecular mass of squirrel monkey PrP was greater than the other forms of primate PrP due to the presence of an extra octapeptide repeat in the N-terminal region (Fig. 1).

### Accumulation of prion seeding activity in prion-exposed primate PrP *Drosophila*

We first determined whether primate PrP *Drosophila* were permissive for mammalian prion propagation. To do so, we exposed *Drosophila* at the larval stage to either vCJD-infected human, classical BSE-infected bovine or CWD-infected white-tailed deer brain homogenate, or respective prion-free normal brain homogenate. After hatching, *Drosophila* were transferred to inoculum-free fly culture tubes. At various time points (≤40 days) during their adult lifespan, groups of *Drosophila* were euthanized, decapitated, and homogenate prepared from the isolated fly heads. These homogenates were used to seed *in vitro* real-time quaking-induced conversion (RT-QuIC) reactions to reveal prion seeding activity.

**Figure 3:**
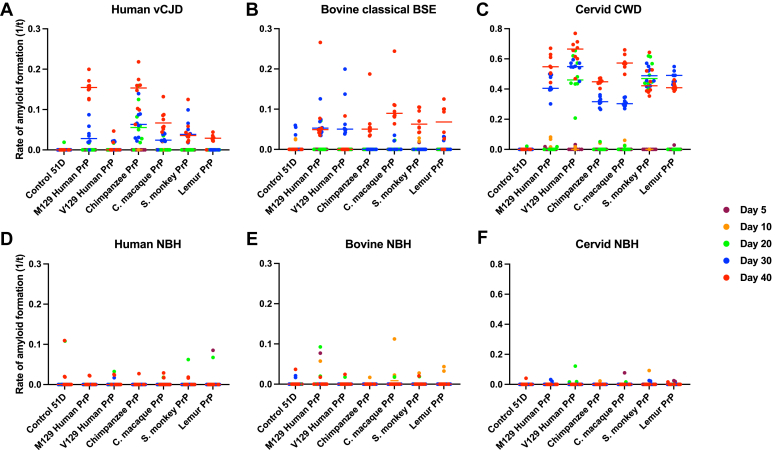
**Accumulation of prion seeding activity in prion-exposed primate PrP *Drosophila.****Elav* x primate PrP *Drosophila* were exposed to human vCJD (*A*), bovine classical BSE (*B*), or cervid CWD (white-tailed deer CWD prion-infected) (*C*), or prion-free human normal brain homogenate (NBH) (*D*), bovine NBH (*E*), or cervid NBH (*F*) at the larval stage. Adult *Drosophila* were collected at the indicated time points after hatching (see *colored key*), and head homogenate was prepared and used as seed in RT-QuIC reactions. Known RT-QuIC positive and negative fly head homogenates were included as controls for the assay (data not shown). The data shown are rate of amyloid formation 1/time (1/t) for each treatment group. Statistical analysis of Figure 3 is shown in Table S1. BSE, bovine spongiform encephalopathy; CWD, chronic wasting disease; vCJD, variant Creutzfeldt-Jakob disease; PrP, prion protein; RT-QuIC, real-time quaking-induced conversion.

The data in Figure 3 show that exposure of primate PrP *Drosophila* to vCJD, classical BSE, or CWD prions led to accumulation of prion seeding activity as the flies aged. Following exposure to vCJD prions (Fig. 3*A*), M129 human PrP *Drosophila* accumulated more prion seeding activity compared to V129 human PrP *Drosophila,* while the level in nonhuman primate PrP *Drosophila* was of the order: chimpanzee > cynomolgus macaque > squirrel monkey > lemur. Chimpanzee PrP *Drosophila* showed the most rapid accumulation of vCJD prion seeding activity. After exposure to classical BSE prions (Fig. 3*B*), human and nonhuman PrP *Drosophila* accumulated similar levels of prion seeding activity and did so with a similar time course. Following exposure to CWD prions (Fig. 3*C*), V129 human PrP *Drosophila* accumulated prion seeding activity more rapidly than M129 human PrP *Drosophila*, while squirrel monkey PrP *Drosophila* accumulated prion seeding activity more rapidly than other nonhuman primate PrP fly lines. Overall, CWD prions induced higher levels of prion seeding activity in primate PrP *Drosophila* than that induced by vCJD or classical BSE prions. Background levels of prion seeding activity were detected in primate PrP *Drosophila* exposed to prion-free control human (Fig. 3*D*), bovine (Fig. 3*E*), or cervid (Fig. 3*F*) brain material. Nontransgenic control 51D *Drosophila* showed background levels of prion seeding activity after exposure to vCJD, classical BSE or CWD prion-infected, or prion-free inoculum (Fig. 3, *A*–*F*). Statistical analysis of Figure 3 is shown in Supporting information Table S1.

### Prion-induced toxicity in primate PrP *Drosophila*

Prion replication in mammalian hosts is associated with prion-induced toxicity (50, 51). Accordingly, we next investigated whether mammalian prion-exposure of primate PrP *Drosophila* led to the development of a neurotoxic phenotype in these flies. To do so, we first performed a negative geotaxis climbing assay using adult *Drosophila* previously exposed to vCJD, classical BSE or CWD prions, or control inoculum, at the larval stage. The locomotor activity of adult *Drosophila* was subsequently assessed and expressed as a performance index.

**Figure 4:**
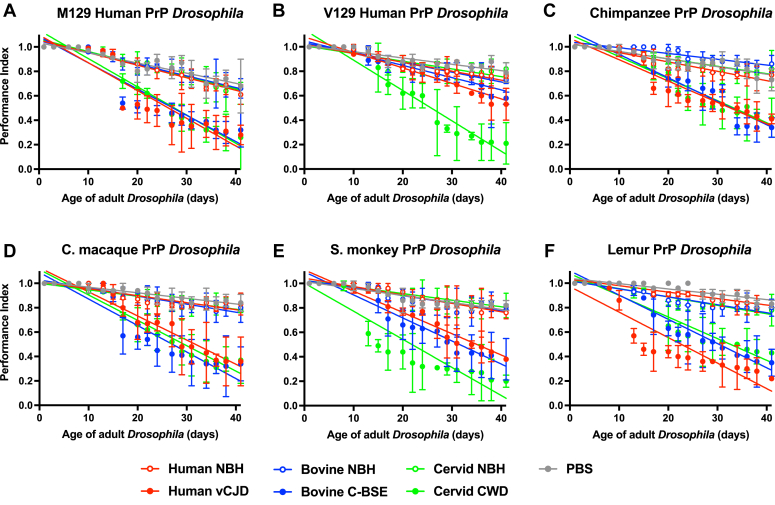
**Accelerated decline of locomotor activity in prion-exposed primate PrP *Drosophila.****Elav* x primate PrP *Drosophila* were exposed to human vCJD, bovine classical BSE or white-tailed deer CWD prion-infected, or prion-free human, bovine or cervid brain material at the larval stage. After hatching, flies were assessed for locomotor activity by a negative geotaxis climbing assay. The mean performance index is shown for three groups of n = 15 flies of each genotype per time point. Statistical analysis of the linear regression plots was performed using an unpaired (two-tailed) Student *t* test. All prion-exposed primate PrP *Drosophila* treatment group plots were significantly different (*p* < 0.05) from their respective prion-free control plots over the whole of the climbing assay time course. M129 human PrP *Drosophila* (*A*), or V129 human PrP *Drosophila* (*B*), chimpanzee PrP *Drosophila* (*C*), cynomolgus macaque PrP *Drosophila* (*D*), squirrel monkey PrP *Drosophila* (*E*) and lemur PrP *Drosophila* (*F*). The mean performance index for control 51D *Drosophila* is shown in Figure S1. Statistical analysis of Figure 4 is shown in Table S2. BSE, bovine spongiform encephalopathy; CWD, chronic wasting disease; PrP, prion protein; vCJD, variant Creutzfeldt-Jakob disease.

The data in Figure 4 demonstrate that adult primate PrP *Drosophila*, exposed to mammalian prions at the larval stage, showed an accelerated decline in climbing ability compared to control treated flies. The accelerated decline in climbing ability became progressively more severe with the increasing age of the prion-exposed flies. M129 human PrP *Drosophila* (Fig. 4*A*) showed an enhanced accelerated decline in climbing ability in response to vCJD and classical BSE prions compared with the responses seen by the V129 human PrP fly line (Fig. 4*B*), although both fly lines showed a similar neurotoxic response to CWD prions. Chimpanzee PrP (Fig. 4*C*) and cynomolgus macaque PrP (Fig. 4*D*) *Drosophila* showed an equal accelerated decline in climbing ability after exposure to vCJD, classical BSE, or CWD prions. Squirrel monkey PrP *Drosophila* (Fig. 4*E*) also showed an accelerated loss of climbing ability after exposure to vCJD, classical BSE, or CWD inoculum but showed the greatest sensitivity to CWD prions. Lemur PrP *Drosophila* (Fig. 4*F*) also showed an accelerated loss of climbing ability after exposure to vCJD, classical BSE, or CWD inoculum but showed the greatest sensitivity to vCJD prions. Supporting information Fig. S1 shows that nontransgenic control 51D *Drosophila* demonstrated no difference in decline in climbing ability after exposure to vCJD, classical BSE, or CWD prion inoculum compared to that seen after exposure to prion-free material. Statistical analysis of Figure 4 and Supporting information Fig. S1 is shown in Supporting information Table S2.

**Figure 5:**
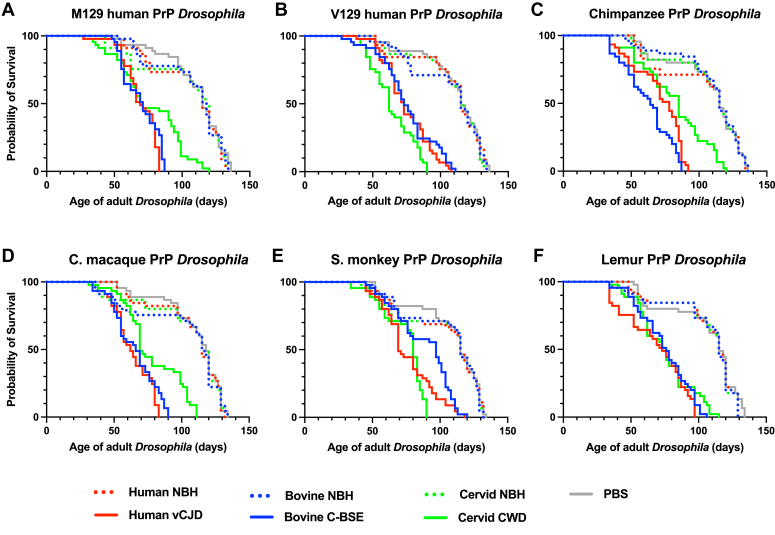
**Accelerated decline of loss of survival of prion-exposed primate PrP *Drosophila.****Elav* x primate PrP *Drosophila* or *Elav* x control 51D *Drosophila* were exposed to human vCJD, bovine classical BSE or white-tailed deer CWD prion-infected, or prion-free human, bovine or cervid brain material, or PBS at the larval stage, respectively. After hatching, the number of surviving flies was recorded three times a week and the data shown as Kaplan–Meier plots. (*A*) M129 human PrP *Drosophila*, or (*B*) V129 human PrP *Drosophila*, (*C*) chimpanzee PrP *Drosophila*, (*D*) cynomolgus macaque PrP *Drosophila*, (*E*) squirrel monkey PrP *Drosophila*, and (*F*) lemur PrP *Drosophila*. The survival curves for control 51D *Drosophila* are shown in Figure S2. Statistical analysis of Figure 5 and Figure S2 is shown in Table S3. BSE, bovine spongiform encephalopathy; CWD, chronic wasting disease; PrP, prion protein; vCJD, variant Creutzfeldt-Jakob disease.

We next assessed the survival of adult primate PrP *Drosophila* after exposure to vCJD, classical BSE, and CWD prions at the larval stage. The data in Figure 5 show that all the primate PrP *Drosophila* fly lines demonstrated a significantly accelerated loss of survival after exposure to each of the different prion inocula compared with exposure to control prion-free inoculum. Supporting information Figure S2 shows that nontransgenic control 51D *Drosophila* demonstrated no difference in rate of survival after exposure to vCJD, classical BSE, or CWD prion inoculum compared with that seen after exposure to prion-free material. Supporting information Table S3 shows median survival times for each fly line in response to each of the different prion inocula. M129 and V129 human PrP *Drosophila* fly lines showed similar median survival times after exposure to classical BSE prion inoculum. After exposure to CWD prions, V129 human PrP *Drosophila* showed a shorter median survival time than the M129 human PrP fly line, and cynomolgus macaque PrP *Drosophila* showed the shortest median survival time compared to the other primate PrP fly lines. Control 51D *Drosophila* showed a similar median survival time after exposure to all inocula.

The survival data described above indicated that vCJD prions propagated equally efficiently in M129 and V129 human PrP *Drosophila*. This contrasts with the transmission of vCJD prions in transgenic mice homozygous for Val129 human PrP which occurs in a restricted manner and is coupled with the emergence of a prion with distinct strain features (52). We therefore identified the prion associated with V129 and M129 human PrP *Drosophila*-passaged vCJD by subsequent transmission in bovine PrP transgenic mice, a validated reporter of the human prion strain (53).

**Figure 6:**
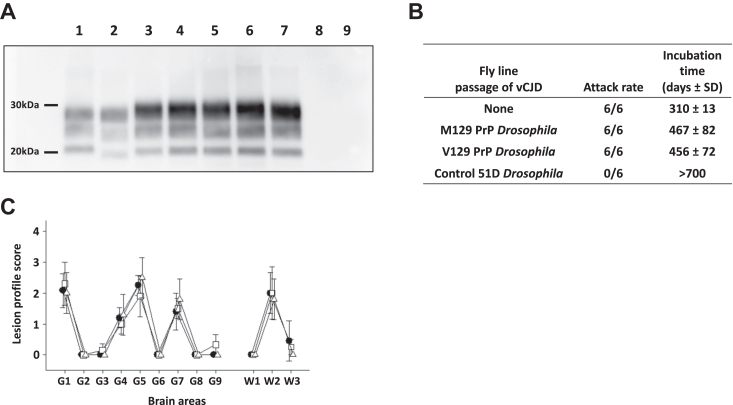
**Authentic vCJD prion strain propagation in human PrP *Drosophila.****Elav* x M129 and *Elav* x V129 human PrP *Drosophila*, and *Elav* x control 51D *Drosophila*, were exposed to vCJD prion inoculum at the larval stage and harvested at 40 days post hatching when head homogenate was prepared from harvested flies and intracerebrally inoculated into bovine PrP transgenic (tgBov) mice. As a control, the original vCJD prion inoculum was also inoculated into tgBov mice. Inoculated mice were euthanized when they showed clinical signs of prion infection or after 700 days for those that did not develop clinical disease. Western blot analysis of inoculated tgBov mouse brains is shown in Figure 6*A*. Mice were considered positive for prion disease when proteinase K-resistant PrP 27–30 was detected in brain tissue by Western blot. Lane 1: sheep scrapie DAW isolate (positive control); lane 2: vCJD isolate (positive control); lane 3: direct transmission of vCJD isolate in tgBov; lanes 4 and 5: vCJD passaged in Met129 human PrP *Drosophila* transmitted to tgBov (representing two different mouse samples); lanes 6 and 7: vCJD passaged in Val129 human PrP *Drosophila* transmitted to tgBov (representing two different mouse samples); and lanes 8 and 9: vCJD passaged in control 51D *Drosophila* transmitted to tgBov (representing two different mouse samples). The attack rate (number of prion-positive mice/total number of mice inoculated) and the incubation time for inoculated mice, which represents the average period from inoculation to euthanasia for each inoculated group of animals, shown in days ± SD, is reported for each treatment group and are shown in Figure 6*B*. Lesion profile analysis of prion-diseased mice was established by scoring the vacuolar changes observed in predefined brain areas and is shown in Figure 6*C*: *filled circle*, vCJD isolate; *open square*, vCJD passaged in Met129 human PrP *Drosophila*; and *open triangle*, vCJD passaged in Val129 human PrP *Drosophila*. The data shown are mean lesion profile scores (three to six mouse brains examined for each isolate) for the following areas of the brain: for gray (G) matter, G1, dorsal medulla nuclei; G2, cerebellar cortex of the folia, including the granular layer, adjacent to the fourth ventricle; G3, cortex of the superior colliculus; G4, hypothalamus; G5, thalamus; G6, hippocampus; G7, septal nuclei of the paraterminal body; G8, cerebral cortex (at the level of G4 and G5); G9, cerebral cortex (at the level of G7); for white (W) matter, W1, cerebellar peduncles; W2, white matter in lateral tegmentum; W3, cerebellar peduncle/internal capsule. PrP, prion protein; vCJD, variant Creutzfeldt-Jakob disease.

The data in Figure 6 show that prion disease was confirmed in these mice by Western blot analysis of mouse brain homogenates (Fig. 6*A* lanes 3–7), which demonstrated PrP 27–30 with a predominance of diglycosylated PrP as was seen in the original inoculum (Fig. 6*A* lane 2). The bovine PrP transgenic mice succumbed to prion disease with the same attack rate and similar incubation times after inoculation with vCJD prions that had been passaged in M129 or V129 human PrP *Drosophila* (Fig. 6*B*). The original vCJD inocula gave the same attack rate of 6/6 mice and an incubation time of 310 ± 13 days which was faster than the *Drosophila* passaged vCJD. Neuropathological analysis of prion-diseased mouse brains showed a lesion profile characteristic of vCJD passaged in bovine PrP transgenic mice (Fig. 6*C*). These combined data show that vCJD human prions propagate with equal efficiency and with authentic prion strain fidelity in both M129 and V129 human PrP *Drosophila*.

Collectively, these data are consistent with the absence of the classical BSE/vCJD transmission barriers associated with V129 human PrP expressed in mammalian species (52, 54) and the absence of the CWD transmission barrier seen in human PrP transgenic mice and cynomolgus macaques (34).

### Human PrP *Drosophila* are susceptible to North American and European CWD prions

To show that the susceptibility of primate PrP *Drosophila* to CWD was not restricted to white-tailed deer prions we next examined the susceptibility of these fly lines to North American and European CWD isolates. We first quantified prion seeding activity in adult human PrP *Drosophila* after exposure to these different CWD inocula at the larval stage. CWD prion-exposed adult human PrP *Drosophila* were harvested at different time points (≤40 days of age) to allow prion seeding activity to be assessed by RT-QuIC on isolated fly head homogenate.

**Figure 7:**
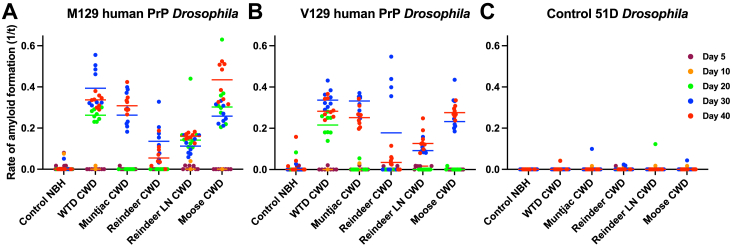
**Prion seeding activity in CWD prion-exposed human PrP *Drosophila.****Elav* x M129 (*A*), *Elav* x V129 (*B*), human PrP *Drosophila,* or *Elav x* control 51D *Drosophila* (*C*) were exposed to CWD-infected North American (white-tailed deer or muntjac) or European (Norwegian reindeer or moose) brain material, or Norwegian reindeer lymph node (LN) material or prion-free control cervid normal brain homogenate (control NBH) at the larval stage. Adult *Drosophila* were collected at the indicated time points after hatching (see *colored key*), and head homogenate was prepared and used as seed in RT-QuIC reactions. Known RT-QuIC positive and negative fly head homogenates were included as controls for the assay (data not shown). The data shown are rate of amyloid formation 1/time (1/t) for each treatment group. Statistical analysis of Figure 7 is shown in Table S4. CWD, chronic wasting disease; PrP, prion protein; RT-QuIC, real-time quaking-induced conversion.

The data in Figure 7 show that M129 (Fig. 7*A*) and V129 (Fig. 7*B*) human PrP *Drosophila* fly lines accumulated prion seeding activity with similar characteristics after exposure to North American and European CWD prion inocula. A similar time course of prion seeding activity accumulation was seen in both fly lines, which became evident at ≥20 days of age, after exposure to each of the different CWD prion inocula. Reindeer CWD prions induced the lowest level of prion seeding activity. Nontransgenic control 51D *Drosophila* (Fig. 7*C*) showed background levels of prion seeding activity after exposure to the various CWD prion inocula or prion-free material. Statistical analysis of prion seeding activity in CWD prion-exposed human PrP *Drosophila* is shown in Supporting information Table S4.

**Figure 8:**
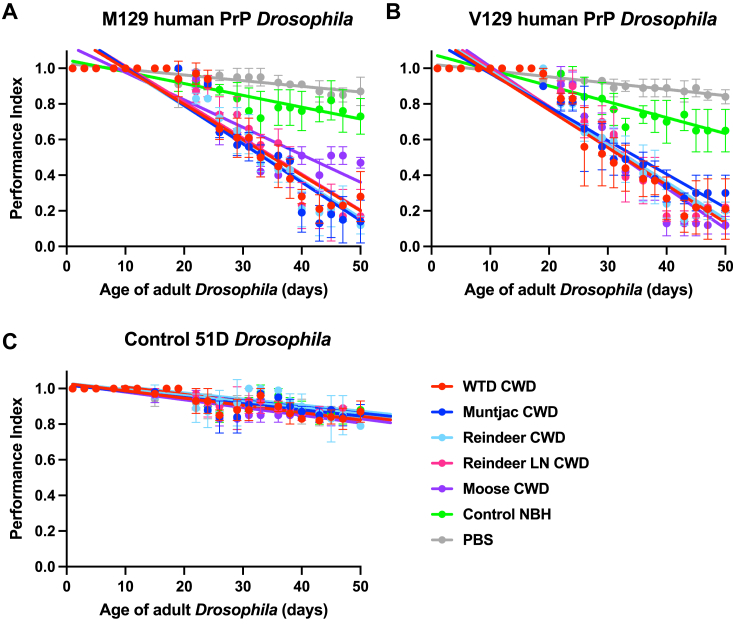
**Accelerated loss of locomotor activity in CWD prion-exposed human PrP *Drosophila.****Elav* x M129 (*A*) or *Elav* x V129 (*B*) human PrP or *Elav* x control 51D (*C*) *Drosophila* were exposed to CWD-infected North American (white-tailed deer or muntjac) or European (Norwegian reindeer or moose) brain material, or Norwegian reindeer lymph node (LN) material or prion-free control cervid normal brain homogenate (control NBH), or PBS, at the larval stage. After hatching, flies were assessed for locomotor activity by a negative geotaxis climbing assay. The mean performance index is shown for three groups of n = 15 flies of each genotype per time point. Statistical analysis of the linear regression plots was performed using an unpaired (two-tailed) Student *t* test. All prion-exposed *Elav* x M129 or *Elav* x V129 human PrP *Drosophila* treatment group plots were significantly different (*p* < 0.05) from their respective prion-free control plots over the whole of the climbing assay time course. Statistical analysis of Figure 8 is shown in Table S5. CWD, chronic wasting disease; PrP, prion protein.

We next assessed the neurotoxic response of M129 human PrP and V129 human PrP *Drosophila* to North American and European CWD prions by a negative geotaxis climbing assay using adult *Drosophila* previously exposed to cervid prions or control inoculum at the larval stage. The data in Figure 8 show that M129 human PrP (Fig. 8*A*) and V129 human PrP (Fig. 8*B*) *Drosophila* demonstrated an accelerated loss of climbing ability that became progressively more severe with age compared with the response to control prion-free inoculum. The prion-induced accelerated loss of climbing ability by both M129 human PrP and V129 human PrP *Drosophila* was similar after exposure to white-tailed deer, muntjac, moose, and reindeer prions. Control 51D *Drosophila* (Fig. 8*C*) showed no difference in decline in climbing ability after exposure to the different CWD prion inocula or prion-free material. Statistical analysis of accelerated loss of locomotor activity in CWD prion-exposed human PrP *Drosophila* is shown in Supporting information Table S5.

**Figure 9:**
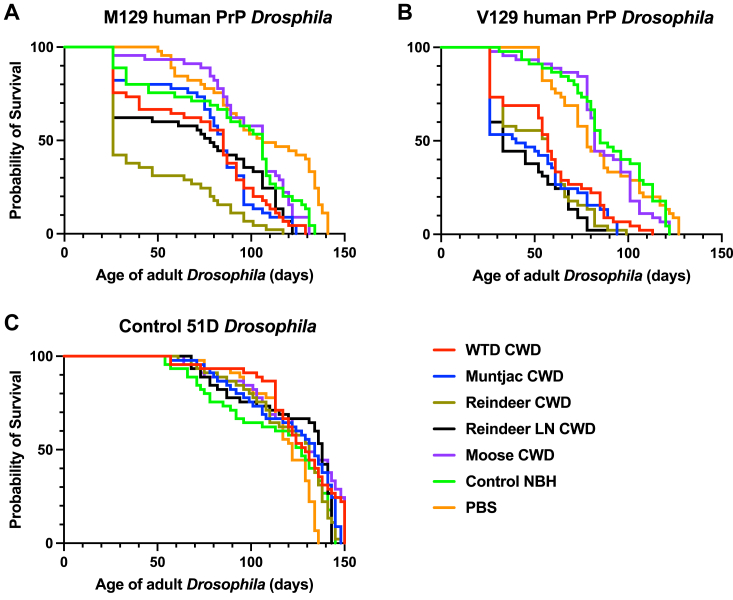
**Accelerated loss of survival of CWD prion-exposed human PrP *Drosophila.****Elav* x M129 (*A*) or *Elav* x V129 (*B*) human PrP or *Elav* x control 51D (*C*) *Drosophila* were exposed to CWD-infected North American (white-tailed deer or muntjac) or European (Norwegian reindeer or moose) brain material, or Norwegian reindeer lymph node (LN) material or prion-free control cervid normal brain homogenate (control NBH), or PBS, at the larval stage. After hatching, the number of surviving flies was recorded three times a week and the data shown as Kaplan–Meier plots. Median survival and statistical analysis of Figure 9 are shown in Table S6. CWD, chronic wasting disease; PrP, prion protein.

We subsequently assessed the survival of adult human PrP *Drosophila* after exposure to North American and European CWD prions at the larval stage. Although human PrP *Drosophila* showed a prion-induced accelerated loss of survival in response to the different CWD inocula, differences and similarities were seen between the M129 and V129 human PrP fly line responses. The data in Figure 9 show that both M129 (Fig. 9*A*) and V129 (Fig. 9*B*) human PrP fly lines showed a significantly accelerated loss of survival in response to CWD inoculum although the response to Norwegian reindeer CWD material was M129 PrP > V129 PrP *Drosophila*. However, the response to North American white-tailed deer and muntjac CWD material was V129 PrP > M129 PrP *Drosophila*. In contrast to these data, both M129 and V129 human PrP fly lines failed to show an accelerated loss of survival after exposure to moose CWD prions. Control 51D *Drosophila* (Fig. 9*C*) showed no difference in loss of survival after exposure to the different CWD prion inocula or prion-free control material. Median survival times of the Kaplan–Meier plots in Figure 9 are shown in Supporting information Table S6.

### Evidence for strain differences between North American CWD and Norwegian moose CWD prions

The failure of the Norwegian moose CWD isolate to induce accelerated loss of survival of human PrP *Drosophila* may reflect the operation of a transmission barrier. Alternatively, this mitigation may be due to low prion titre of the moose CWD inoculum. To exclude the latter possibility, we determined the relative prion titre of the moose CWD inoculum with that of the reindeer CWD isolate, since the latter did cause accelerated loss of survival of the V129 human PrP fly line. Accordingly, we analyzed the locomotor ability, prion seeding activity, and survival of adult cervid PrP *Drosophila* and V129 human PrP *Drosophila* after exposure to a 10^−2^ dilution series of reindeer or moose CWD inoculum at the larval stage.

**Figure 10:**
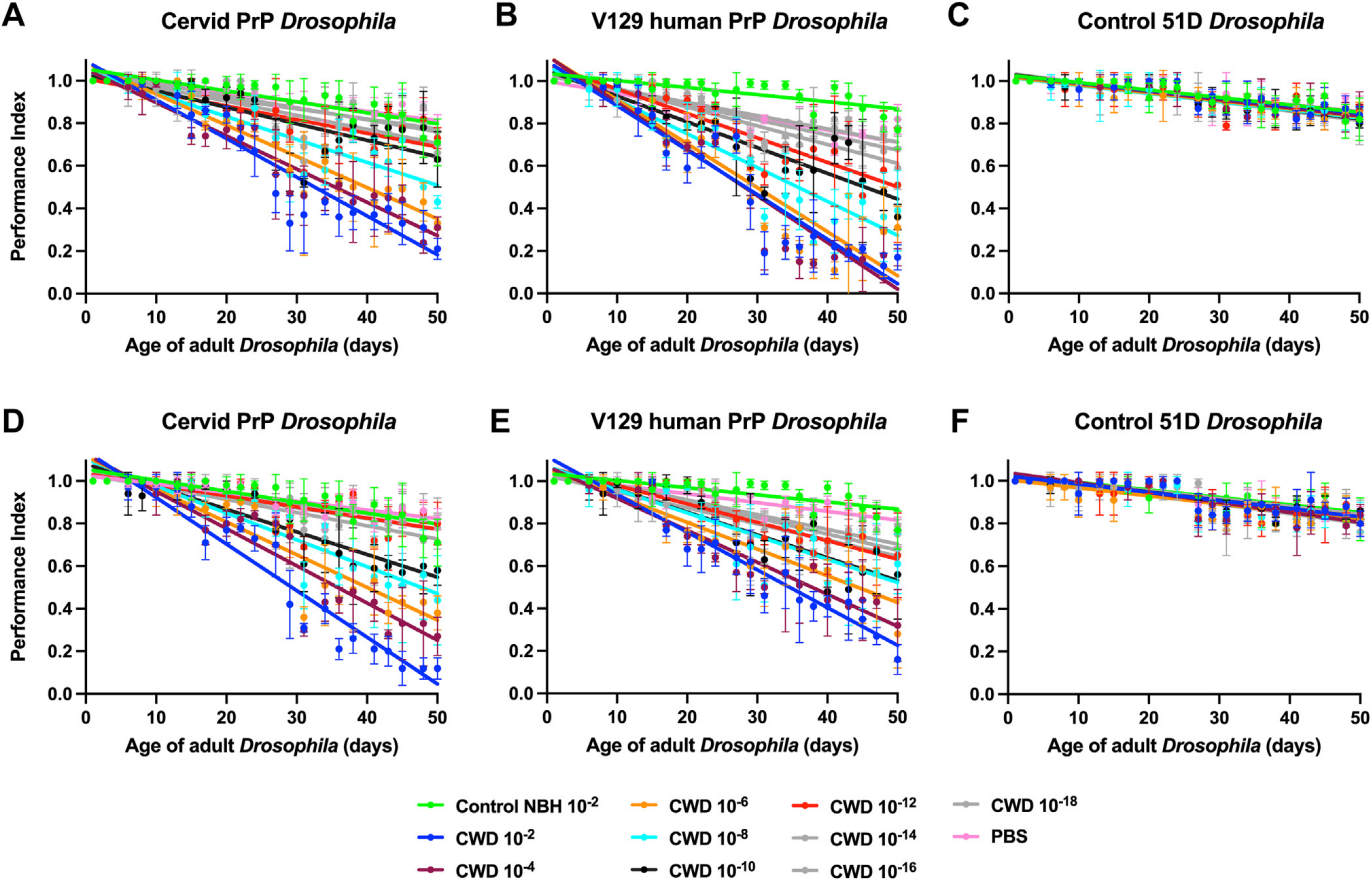
**Accelerated loss of locomotor activity induced by reindeer or moose CWD prions.***Elav* x cervid PrP (*A*) and (*D*), *Elav* x V129 human PrP (*B*) and (*E*), or *Elav* x control 51D (*C*) and (*F*) *Drosophila* were exposed to a 10^−2^ dilution series of European (Norwegian reindeer or moose) CWD-infected brain material, or a 10^−2^ dilution of prion-free control cervid normal brain homogenate (control NBH), or PBS, at the larval stage. After hatching, flies were assessed for locomotor activity by a negative geotaxis climbing assay. The mean performance index is shown for three groups of n = 15 flies of each genotype per time point. Statistical analysis of the linear regression plots was performed using an unpaired (two-tailed) Student *t* test. All prion-exposed *Elav* x cervid PrP and *Elav* x V129 human PrP *Drosophila* treatment group plots were significantly different (*p* < 0.05) from their respective prion-free control plots over the whole of the climbing assay time course. Reindeer CWD prions (*A*-*C*) and moose CWD prions (*D*-*F*). Statistical analysis of Figure 10 is shown in Table S7. Day 50 mean performance index data from Figure 10 are shown plotted against the 10^−2^ dilution series of the relevant CWD prion inoculum in Fig. S3. CWD, chronic wasting disease; PrP, prion protein.

The data in Figure 10 show that adult cervid PrP *Drosophila* (Fig. 10, *A* and *D*) and V129 human PrP *Drosophila* (Fig. 10, *B* and *E*) both showed an accelerated loss of locomotor ability after exposure to an extensive range of dilutions of both the reindeer or moose CWD inoculum, compared to control-treated flies. In both cases, the accelerated loss of locomotor ability decreased with increasing dilution of inoculum. Control 51D *Drosophila* (Fig. 10, *C* and *F*) showed no difference in their response to the dilution series of either CWD inoculum compared to the response seen after exposure to control prion-free material. Statistical analysis of accelerated decline of locomotor activity in prion-exposed *Drosophila* is shown in Supporting information Table S7. The data in Supporting information Fig. S3 show the mean performance index (±SD) for day 50 of the locomotor assay (Fig. S3, *A*–*C* reindeer CWD prions, and Fig. S3, *D*–*F* moose CWD prions), in order to highlight the titration effect seen in Figure 10.

**Figure 11:**
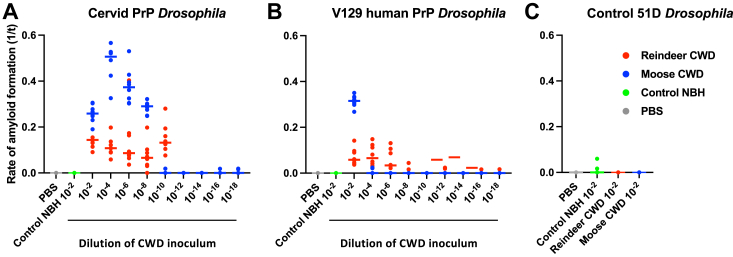
**Differences in prion seeding activity accumulation induced by Norwegian reindeer or moose CWD inoculum.***Elav* x cervid PrP (*A*), *Elav* x V129 human PrP (*B*), or *Elav* x control 51D (*C*) *Drosophila* were exposed to a 10^−2^ dilution series of European (Norwegian reindeer or moose) CWD-infected brain material, or 10^−2^ prion-free control cervid normal brain homogenate (control NBH), or PBS, at the larval stage. Adult *Drosophila* were collected at 40 days post hatching and head homogenate was prepared and used as seed in RT-QuIC reactions. The data shown are rate of amyloid formation 1/time (1/t) for each treatment group. Statistical analysis of Figure 11 is shown in Table S8. CWD, chronic wasting disease; PrP, prion protein; RT-QuIC, real-time quaking-induced conversion.

We next assessed the titre of Norwegian reindeer and moose CWD inoculum by accumulation of prion seeding activity in adult cervid PrP *Drosophila* and V129 human PrP *Drosophila* as shown by the data in Figure 11. Prion seeding activity was detected in adult cervid PrP *Drosophila* (Fig. 11*A*) after exposure to reindeer CWD dilutions between 10^−2^ and 10^−10^ and after exposure to moose CWD dilutions between 10^−2^ and 10^−8^, at the larval stage. However, in adult V129 human PrP *Drosophila* (Fig. 11*B*) prion seeding activity was detected after exposure to reindeer CWD dilutions between 10^−2^ and 10^−8^, but only after exposure to moose CWD inoculum at a 10^−2^ dilution, at the larval stage. Control 51D *Drosophila* (Fig. 11*C*) failed to accumulate prion seeding activity after exposure to either Norwegian reindeer or moose CWD inoculum. Statistical analysis of prion seeding activity in prion-exposed cervid or human PrP *Drosophila* is shown in Supporting information Table S8.

**Figure 12:**
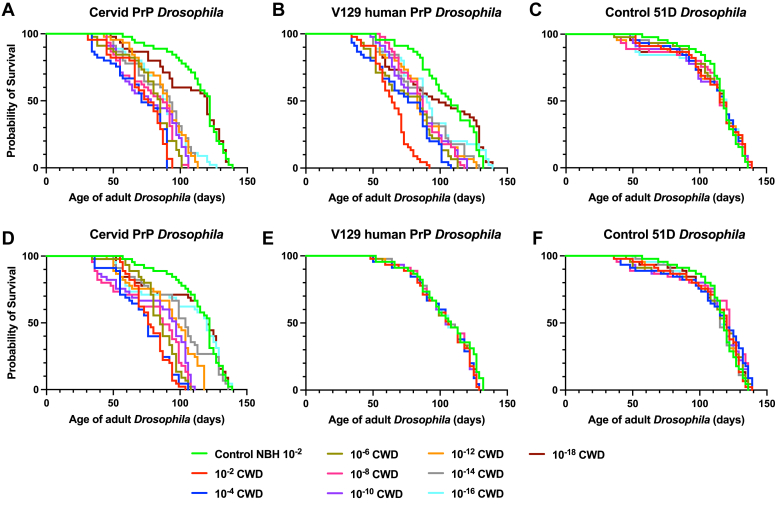
**Moose CWD prions do not affect survival of V129 human PrP *Drosophila.****Elav* x cervid PrP (*A*) and (*D*), *Elav* x V129 human PrP (*B*) and (*E*), or *Elav* x control 51D (*C*) and (*F*) *Drosophila* were exposed to a 10^−2^ dilution series of European (Norwegian reindeer or moose) CWD-infected brain material, or 10^−2^ dilution of prion-free control cervid normal brain homogenate (control NBH) at the larval stage. After hatching, the number of surviving flies was recorded three times a week and the data shown as Kaplan–Meier plots. Reindeer CWD prions (*A*–*C*) and moose CWD prions (*D*–*F*). Statistical analysis of Figure 12 is shown in Table S9*A* and Table S9*B*. CWD, chronic wasting disease; PrP, prion protein.

The data in Figure 12 show that adult cervid PrP *Drosophila* (Fig. 12, *A* and *D*) and V129 human PrP *Drosophila* (Fig. 12, *B* and *E*) generated a median survival time of 122 days and 108 days, respectively, after exposure to prion-free cervid brain material at the larval stage. In contrast, the median survival time of both fly lines was significantly reduced after exposure to dilutions in the range of 10^−2^ to 10^−14^ of reindeer CWD inoculum. The reduction in median survival time compared to the control value was decreased with increasing dilution of reindeer CWD inoculum. However, the data in Figure 12 also show that while the median survival time of cervid PrP *Drosophila* was significantly reduced after exposure at the larval stage to moose CWD inoculum in the dilution range of 10^−2^ to 10^−14^ there was no similar reduction in median survival time of V129 human PrP *Drosophila* after exposure to any of the dilutions of moose CWD inoculum. The control 51D fly line (Fig. 12, *C* and *F*) showed no accelerated loss of survival when exposed to the dilution range of reindeer or moose CWD inocula compared to prion-free control inoculum. Supporting information Table S9*A* (reindeer CWD) and Table S9*B* (moose CWD) show the median survival time in days and the statistical analysis of the Kaplan-Meier plots in Figure 12.

Collectively, these data show that the Norwegian reindeer and moose CWD inoculum used here had a similar relative prion titre but showed differential effects when propagated in cervid PrP and V129 human PrP *Drosophila.*

## Discussion

Evidence for the zoonotic behavior of animal prions was first provided by epidemiological data that showed the temporal relationship between the classical BSE epizootic and subsequent emergence of vCJD in humans (27, 28, 29, 30). The human transmission barrier for classical BSE has since been modeled *in vivo* in nonhuman primates and human PrP transgenic mice. Cynomolgus macaques inoculated with classical BSE prions developed neuropathology like that seen in humans with vCJD (55, 56). The influence of human PrP codon 129 on susceptibility to classical BSE was modeled by inoculation of human PrP transgenic mice (57), which confirmed susceptibility of the M129 PrP variant and that the V129 variant was associated with resistance. In addition to these studies, *in vitro* PMCA and RT-QuIC successfully modeled misfolding of human PrP induced by classical BSE PrPSc. The application of epidemiological studies together with *in vivo* and *in vitro* modeling methodologies with the known prion zoonotic classical BSE established a reference framework to assess the zoonotic potential of other animal prion diseases. For example, in the case of CWD, there is no reported epidemiological evidence that links cervid prion disease to increased cases of a known prion disease, or of any other neurodegenerative condition, in humans (58, 59, 60, 61). When modeled in nonhuman primates, squirrel monkeys were found to be susceptible to CWD prions (34), whereas cynomolgus macaques which are considered evolutionary closer to humans (45, 62) and therefore a more relevant animal model, showed resistance (34, 63, 64). In addition, to date all but one study with human PrP transgenic mice inoculated with CWD material failed to demonstrate cervid prion transmission (34, 65). Modeling of the zoonotic potential of CWD using *in vitro* cell-free conversion assays have suggested human PrP can act as a substrate for CWD prion propagation (66). Despite their lack of consensus, these studies have collectively suggested a robust transmission barrier limits the zoonotic potential of CWD prions in humans. However, the current experimental methodologies used to support this prediction have drawbacks and limitations. For example, *in vivo* prion bioassays with mammalian hosts have significant practical difficulties, are subject to increasing ethical debate, and in the case of nonhuman primates are typically low-power experiments that lack same-species confirmatory secondary passage. In addition, *in vitro* PrP conversion assays are usually performed in the absence of a cellular or molecular milieu and therefore lack potential regulators of prion transmission barriers. To circumvent these issues, new experimental systems are required to help define the extent and strength of the human prion transmission barrier with respect to animal prion diseases such as CWD.

Here, we investigated whether PrP transgenic *Drosophila*, an already established mammalian prion bioassay host (42, 43, 44), was suitable to model the human prion transmission barrier. Surprisingly, our studies showed that *Drosophila* transgenic for human or nonhuman primate PrP did not recapitulate known prion transmission barriers seen in mammalian hosts as evidenced by several observations. Firstly, both M129 and V129 human PrP transgenic *Drosophila* were found to be equally permissive for classical BSE and vCJD prion propagation. This was shown by the similar accelerated loss of survival by both fly lines after exposure to either classical BSE or vCJD prions; the similar incubation times for bovine PrP transgenic mice inoculated with M129 or V129 human PrP *Drosophila*-passaged vCJD; and the observation of authentic vCJD prion strain propagation in both fly lines. These observations contrast with studies in mammalian hosts since expression of the V129 PrP variant in humans is associated with resistance to clinical vCJD after exposure to BSE prions (54), and mice transgenic for Val129 human PrP are severely resistant to classical BSE infection and are sufficiently restricted to infection with vCJD prions to allow emergence of a distinct prion strain (52). Secondly, after exposure to CWD prions, *Drosophila* transgenic for primate PrP, including human and cynomolgus macaque, showed progressive accumulation of prion seeding activity accompanied by an increasingly severe neurotoxic phenotype that culminated in accelerated loss of their survival. These data also contrast with prion transmission studies in mammalian hosts since human PrP transgenic mice and cynomolgus macaques show significant resistance to CWD prions (34, 63, 64, 65). In this light, our studies here suggest that PrP transgenic *Drosophila* lack prion transmission barriers seen in mammalian hosts and as such act as a “universal acceptor” of mammalian prions. Consequently, PrP transgenic *Drosophila* would appear to be unsuitable, in their present format, for use in the assessment of the zoonotic potential of animal prions.

The mammalian cross-species prion transmission barrier has been attributed principally to the lack of amino acid sequence homology between donor and host PrP (17, 18). Another species, namely the bank vole (*Myodes glareolus*) is susceptible to prions from a diverse range of species, which suggests prion transmission barriers are also mitigated in this rodent host (67). In the case of the bank vole, prion promiscuity is attributed to a broad interspecies compatible bank vole PrP amino acid sequence. This was clearly not the case here since we generated *Drosophila* transgenic for the same amino acid sequence of PrP expressed in natural mammalian hosts that are associated with resistance to prion propagation, on specific occasions. In this context, our observations showed strikingly that transposition of PrP amino acid sequence between two disparate hosts, in this case those phylogenetically separated by millions of years of divergent evolution, leads to mitigation of a major regulator of mammalian prion propagation. Accordingly, our studies argue against lack of PrP homology as the principal driver of the cross-species prion transmission barrier (17, 18). However, a major influence upon the effectiveness of the human prion transmission barrier is the identity of the prion strain attempting to undergo cross-species propagation (19, 20). It could be argued, therefore, that the reason for our observed lack of transmission barriers in PrP transgenic *Drosophila* was because different prion strains were used compared to those used in experiments with mammalian hosts that do show transmission barriers. Potentially this was possible in the CWD experiments performed here since we used cervid prion isolates with undefined prion strain identity. However, this was certainly not the case with the bovine and human inoculum used here since classical BSE and vCJD prions constitute a single-defined prion strain (29, 68, 69).

Several reasons can be proposed for the absence of an operational cross-species prion transmission barrier in *Drosophila*. First, the conformational plasticity of mammalian PrP expressed in *Drosophila* may be greater than in a natural host, which may arise through differences in protein glycosylation. *Drosophila* can synthesize N-linked glycans with core structures similar to other eukaryotes (70) but lack complex carbohydrate structures that contain N-acetylglucosamine, mannose, galactose, and terminal sialic acid residues (71) seen on proteins, including PrP, in mammalian species. Sialic acid residues may play a regulatory role in PrP misfolding since *in vitro* PMCA with desialylated PrPC proceeds at an amplified rate with an apparent alleviation of the species barrier (72). As such, intermediates of PrP misfolding may form more readily when the protein is expressed in *Drosophila*. Second, *Drosophila* may lack the presence, or have reduced activity levels, of proteostatic mechanisms that promote efficient removal of protein aggregates in order to protect neurons from proteotoxicity. We have previously shown that overexpression of the Hsp70 disaggregase complex in PrP transgenic *Drosophila* enhances the clearance of mammalian prions (73). Third, *Drosophila* may possess cofactors that are particularly permissive for mammalian prion replication (74) or alternatively, not express cofactors that facilitate the prion transmission barrier. Since PrP transgenic *Drosophila* do support authentic mammalian prion strain propagation (42), any cofactors that do regulate the transmission barrier would have to be distinct from those that ensure maintenance of prion strain fidelity.

It will be important to substantiate our observed lack of prion transmission barriers in PrP transgenic *Drosophila* through further study. This can be addressed through the generation of new *Drosophila* fly lines that are transgenic for PrP from mammalian species with known resistance to prion infection (*e.g.,* porcine, rabbit, horse, or dog) (75) and to determine their susceptibility to different prion sources. Also, it will be important to determine the identity of those prion strains that propagate in primate PrP transgenic *Drosophila* following CWD prion infection. Retention of cervid prion strain identity following these transmissions, as shown here with passage of vCJD prions in V129 human PrP transgenic *Drosophila*, would provide further evidence for lack of transmission barriers in PrP transgenic *Drosophila*. This would be particularly important in the case of European moose prion transmission in V129 human PrP *Drosophila,* where there was evidence of a transmission barrier. Furthermore, it should be noted that classical BSE and vCJD prion-exposed V129 human PrP *Drosophila* showed a reduced locomotor defect compared to M129 human PrP *Drosophila* exposed to the same inocula. This may also imply that V129 human PrP *Drosophila* have the basis of a transmission barrier, one that is overwhelmed by efficient prion propagation because of the magnitude or topological expression of the PrP transgene. It would therefore be interesting to determine if a different level or different neuronal distribution of transgenic PrP expression in *Drosophila* would be accompanied by the presence of an effective prion transmission barrier for distinct prion strains. This could be achieved with different driver fly lines for induction of PrP expression in *Drosophila*. It is known that the level of PrP transgene expression can affect the level of cross-species prion transmission.

In summary, our studies here have shown that prion transmission barriers that are evident in mammalian species are not present, or as apparent, in PrP transgenic *Drosophila*. We have previously shown that *Drosophila*, a normally PrP-null insect host, rendered transgenic solely for mammalian PrP, are significantly more sensitive for the detection of mammalian prions than a mammalian host, namely PrP transgenic mice. The high sensitivity of *Drosophila* for mammalian prions may arise because of one or more of the following reasons: *Drosophila* may provide a more favorable environment for prion replication, accumulation, and spread than is found in mice, either through enhanced prion formation and/or reduced prion clearance or both of these effects; a broader range of PrPSc conformers may be infectious in *Drosophila*; the exposure of *Drosophila* to prions at an earlier stage of development compared with that of mice may fortuitously render the invertebrate host more sensitive to prion replication and prion-induced toxicity during adulthood. We propose that PrP transgenic *Drosophila*, with their apparent lack of prion transmission barriers, could support such zoonotic prion studies in mammalian species through their use as a surrogate second-passage host for the relatively rapid, ultrasensitive, detection of mammalian prion infectivity. For example, cynomolgus macaque PrP transgenic *Drosophila* could be used to bioassay tissues and fluids from CWD-exposed cynomolgus macaques. It may be the case that the cause of the sensitive detection of prions by PrP transgenic *Drosophila* is also responsible, either wholly or in part, for the absence of prion transmission barriers in this invertebrate host. In this scenario, PrP transgenic *Drosophila* can be used not only for the relatively rapid detection of low levels of *bona fide* mammalian prion infectivity but also as a new experimental host to probe the basis of prion transmission barriers.

## Experimental procedures

### Generation of primate PrP transgenic *Drosophila*

*Drosophila* transgenic for primate PrP were generated by pUASTattB/PhiC31-mediated site-specific transformation (46). The human PrP transgenes comprised DNA encoding an insect secretion signal peptide at the 5′ end (76) followed by DNA encoding the M129 or V129 variants of mature-length human PrP (GenBank accession number AF076976, amino acid residues 23–230) and DNA encoding the human PrP glycosylphosphatidylinositol anchor signal sequence (amino acid residues 231–253) at the 3' end. The human PrP transgenes were prepared by a two-step PCR as previously described (43). The first PCR used brain material from mice transgenic for M129 (Tg650) (77) or V129 (Tg362) (78) human PrP as substrate and oligonucleotide primers HuPDF1: 5′ CCA TCT TCT GGC TGC TCA GAC CTT CGC CCA GAA GAA GCG CCC GAA GCC TGG AG 3′ and HuPDR1: 5′ GTC CGC TCG AGT CTA GAT CAT CCC ACT ATC AGG AAG ATG 3′. A second PCR was carried out using the 765 bp product of the first PCR reaction as substrate and oligonucleotide primers PD2F: 5′-GGC GAA TTC ATG GCG AGC AAA GTC TCG ATC CTT CTC CTG CTA ACC GTC CAT CTT CTG C-3′ and HuPDR1 already listed above. The following species forms of nonhuman primate PrP, with their respective GenBank DNA accession number shown in brackets, were used: chimpanzee (U08296); cynomolgus macaque (U08298); squirrel monkey (U08310); or lemur (DQ014540). The nonhuman primate PrP transgenes, which were synthesized commercially, (Genewiz) comprised DNA encoding an insect secretion signal peptide at the 5′ end (76) followed by DNA encoding mature nonhuman primate PrP (amino acid residues 23–230 for chimpanzee and cynomolgus macaque; amino acid residues 23–237 for squirrel monkey; and amino acid residues 23–231 for gray mouse lemur) and DNA encoding the species-specific PrP glycosylphosphatidylinositol anchor signal sequence at the 3' end. The human and nonhuman primate PrP transgenes contained *EcoR1* and *Xho1* restriction sites at the 5′ and 3′ end, respectively, that allowed directional cloning into the *Drosophila* transgenesis vector pUASTattB. The 3′ end of the PrP transgenes contained a stop codon ahead of the *Xho1* restriction site. The PrP transgenes were subsequently ligated into the transgenesis vector pUASTattB and rescued by transformation in DH5α bacteria. pUASTattB primate PrP plasmid DNA was isolated from transformed bacteria by an alkaline lysis method using the Qiagen maxiprep kit and the PrP construct insert verified by DNA sequence analysis. Site-specific transformation of the pUASTattB-PrP constructs into the red fluorescent protein (RFP)-free 51D variant fly line derived from (y[1] M{vas-int.Dm}ZH-2A w[∗]; M{3 x P3-RFP.attP}ZH-51D) was performed by the Department of Genetics, Cambridge University. F1 flies were balanced, the inserted PrP transgenes were verified by DNA sequence analysis, and viable fly lines were maintained as balanced stocks by conventional fly crosses. *Drosophila* transgenic for WT (S138) white-tailed deer PrP have been described previously (44). The following fly lines were obtained from the Department of Genetics, University of Cambridge, UK: *Elav-GAL4* (P{w[+mW.hs] = GawB}elav[C155]); and the RFP-free 51D variant fly line. All fly lines were raised on standard cornmeal media at 25 ˚C and maintained at low to medium density. Flies were used in the assays described below or harvested at various time points and then frozen at −80 ˚C until required.

### Western blot of *Drosophila* head homogenate

Primate PrP *Drosophila* head homogenates (two fly head equivalents per track for nonhuman primate PrP transgenic *Drosophila* and ten fly head equivalents per track for the human primate PrP transgenic *Drosophila*) were prepared for SDS-PAGE and Western blot as described in detail previously (79) except that the nitrocellulose membranes were probed with a 1:2000 dilution of anti-PrP mAb Sha31 (80). PrP bands were detected by enhanced chemiluminescence and captured by a Chemidoc MP Imaging System (Bio-Rad).

### Prion inoculation of *Drosophila*

Prion inocula consisted of vCJD-infected (sample T00047807) or control MM129 human brain homogenate (supplied by the CJD Surveillance Unit) prepared in PBS pH 7.4. Classical BSE-infected (sample SE2015/0001) or control bovine brain homogenate (supplied by the APHA) prepared in PBS. Cervid brain homogenate, prepared in PBS, of cerebral cortex tissue from confirmed cases of North American experimental CWD in white-tailed deer or muntjac (81, 82) and Norwegian natural CWD in moose (17 CD 11399) or reindeer (17 CD 11087), or reindeer parotid lymph node (17 CD 11087). Confirmed CWD-free cervid brain tissue or PBS was used as control inocula for CWD-positive cervid material. *Drosophila* at the larval stage of development were exposed to either 10^−2^ (1% w/v) final concentration of vCJD-infected or control MM129 human brain homogenate; 10^−2^ (1% v/v) final concentration of classical BSE-infected or (1% w/v) for control bovine brain homogenate; 10^−2^ (1% w/v) final concentration of CWD-infected reindeer parotid lymph node or negative cervid brain homogenate; 10^−2^ (1% w/v) final concentration or a 10^−2^ dilution series as shown in Figure 10, Figure 11, Figure 12 of CWD-infected reindeer or moose brain homogenate, prion-free control cervid brain homogenate, or 1× PBS. Two hundred and fifty microliters of each sample prepared in PBS were added to the top of the cornmeal that contained third instar *Drosophila* larvae in 3-inch plastic vials. Following eclosion (*i.e.,* hatching) flies were transferred to fresh nontreated vials.

### Detection of prion seeding activity by RT-QuIC

RT-QuIC with syrian hamster recombinant PrP (rhaPrP: amino acids 90–231) as substrate was performed as described previously (81). Seed was *Drosophila* head homogenate prepared as described above and diluted 1:10 in 0.1% SDS (83). The RT-QuIC threshold was set at 5 SD above the mean of the initial five readings. The inverse of the time when the reaction reached the threshold (1/time to threshold) was then used to determine the amyloid formation rate. Statistical analyses were run in Prism v9, GraphPad Software, www.graphpad.com.

### *Drosophila* negative geotaxis climbing assay

The locomotor ability of flies was assessed in a negative geotaxis climbing assay initiated with 45 (3 × n = 15) age-matched, premated female flies in each treatment group as previously described (42). The mean performance index ± SD at individual time points for each treatment group was plotted as a regression line.

### *Drosophila* survival assay

Survival of *Drosophila* was assessed and recorded as previously described (43). Survival curves are shown as Kaplan–Meier plots.

### Western blot detection of abnormal PrP

Proteinase K-resistant abnormal PrP (PrP 27–30) was extracted from prion-diseased mouse brains performed as described previously (84). Immunodetection was performed with PrP-specific mAb Sha31 (1 μg/ml), which recognises the amino acid sequence YEDRYYRE (80). PrP bands were detected by enhanced chemiluminescence and captured by a Chemdoc XRS Imager (BioRad).

### Strain typing of human vCJD prions

Human vCJD prions were strain typed in mice that expressed bovine PrP (tgBov-tg110) (85). Groups of 6- to 10-week-old female bovine PrP transgenic mice (tgBov-tg110) (*n* ≤ 6) were anesthetized and inoculated intracerebrally into the right parietal lobe using a 25-gauge disposable hypodermic needle with 20 μl of a 10% vCJD-positive human brain homogenate or 20 μl of diluted adult *Drosophila* head homogenate (to give approximately two fly-head equivalents per mouse). Inoculated mice were observed daily, and their neurological status was assessed weekly. Mice were euthanized when clinically progressive prion disease was evident. In animals where no clinical signs were observed, mice were euthanized at the end of their natural lifespan (>700 days) and in those cases the reported incubation times corresponded to the survival time observed in at least three out of the six mice. Brain tissue (cerebral cortex) was collected from euthanized mice for neuropathological analysis.

### Lesion profiling

Vacuolar brain lesion profile scores on prion-diseased mouse brain tissue were established following the method described by Fraser and Dickinson (86).

### Statistical analysis

Statistical analysis of the prion seeding activity was assessed by the Mann–Whitney or Wilcoxon test to generate *p* values (those <0.05 were considered significant) by comparing the median of the CWD-exposed sample rates to the median of the control treatment rates. Statistical analysis of the negative geotaxis climbing assay data was performed by the unpaired (two-tailed) Student’s *t* test. Statistical analysis of median survival times was carried out using Kaplan–Meier statistics and differences between them were analyzed by the Log-rank (Mantel–Cox) test method. All statistical analyses were performed using Prism v9, GraphPad Software.

### Ethics statement

All animal experiments were performed in compliance with institutional and French national guidelines and in accordance with the European Community Council Directive 86/609/EEC. The animal experiments that are part of this study (national registration 01734.01) were approved by the local ENVT ethic committee. Mouse inoculations were performed under anaesthesia (isoflurane). Mice that displayed clinical signs were anesthetized with isoflurane before sacrifice using CO_2_ inhalation.

## Data availability

All data are included in this manuscript.

## Supporting information

This article contains Supporting information.

## Conflict of interest

The authors declare that they have no conflicts of interest with the contents of this article.

## References

[1] Prusiner S.B. (2004).

[2] Prusiner S.B. (1982). Novel proteinaceous infectious particles cause scrapie. Science.

[3] Castilla J., Saa P., Hetz C., Soto C. (2005). In vitro generation of infectious scrapie prions. Cell.

[4] Deleault N.R., Harris B.T., Rees J.R., Supattapone S. (2007). Formation of native prions from minimal components in vitro. Proc. Natl. Acad. Sci. U. S. A..

[5] Legname G., Baskakov I.V., Nguyen H.O., Riesner D., Cohen F.E., DeArmond S.J. (2004). Synthetic mammalian prions. Science.

[6] Legname G., Nguyen H.O., Baskakov I.V., Cohen F.E., Dearmond S.J., Prusiner S.B. (2005). Strain-specified characteristics of mouse synthetic prions. Proc. Natl. Acad. Sci. U. S. A..

[7] Wang F., Wang X., Yuan C.G., Ma J. (2010). Generating a prion with bacterially expressed recombinant prion protein. Science.

[8] Erana H., Diaz-Dominguez C.M., Charco J.M., Vidal E., Gonzalez-Miranda E., Perez-Castro M.A. (2023). Understanding the key features of the spontaneous formation of bona fide prions through a novel methodology that enables their swift and consistent generation. Acta Neuropathol. Commun..

[9] Cullié J., Chelle P.L. (1936). Pathologie animale : la maladie dite de la tremblante du mouton est-elle inoculable?. Comptes rendus de l'Académie des Sci. D.

[10] Pattison I.H., Gordon W.S., Millson G.C. (1959). Experimental production of scrapie in goats. J. Comp. Pathol..

[11] Gajdusek D.C., Gibbs C.J., Alpers M. (1966). Experimental transmission of a Kuru-like syndrome to chimpanzees. Nature.

[12] Gibbs C.J., Gajdusek D.C., Asher D.M., Alpers M.P., Beck E., Daniel P.M. (1968). Creutzfeldt-Jakob disease (spongiform encephalopathy): transmission to the chimpanzee. Science.

[13] Hill A.F., Joiner S., Linehan J., Desbruslais M., Lantos P.L., Collinge J. (2000). Species-barrier-independent prion replication in apparently resistant species. Proc. Natl. Acad. Sci. U. S. A..

[14] Race R., Chesebro B. (1998). Scrapie infectivity found in resistant species. Nature.

[15] Kimberlin R.H., Walker C.A. (1978). Evidence that the transmission of one source of scrapie agent to hamsters involves separation of agent strains from a mixture. J. Gen. Virol..

[16] Moreno J.A., Telling G.C. (2017). Insights into mechanisms of transmission and pathogenesis from transgenic mouse models of prion diseases. Methods Mol. Biol..

[17] Scott M., Foster D., Mirenda C., Serban D., Coufal F., Walchli M. (1989). Transgenic mice expressing hamster prion protein produce species-specific scrapie infectivity and amyloid plaques. Cell.

[18] Prusiner S.B., Scott M., Foster D., Pan K.M., Groth D., Mirenda C. (1990). Transgenetic studies implicate interactions between homologous PrP isoforms in scrapie prion replication. Cell.

[19] Asante E.A., Linehan J.M., Desbruslais M., Joiner S., Gowland I., Wood A.L. (2002). BSE prions propagate as either variant CJD-like or sporadic CJD-like prion strains in transgenic mice expressing human prion protein. EMBO J..

[20] Scott M.R., Will R., Ironside J., Nguyen H.O., Tremblay P., DeArmond S.J. (1999). Compelling transgenetic evidence for transmission of bovine spongiform encephalopathy prions to humans. Proc. Natl. Acad. Sci. U. S. A..

[21] Collinge J., Clarke A.R. (2007). A general model of prion strains and their pathogenicity. Science.

[22] Gibbs C.J., Gajdusek D.C. (1973). Experimental subacute spongiform virus encephalopathies in primates and other laboratory animals. Science.

[23] Gibbs C.J., Gajdusek D.C. (1972). Transmission of scrapie to the cynomolgus monkey (Macaca fascicularis). Nature.

[24] Comoy E.E., Mikol J., Luccantoni-Freire S., Correia E., Lescoutra-Etchegaray N., Durand V. (2015). Transmission of scrapie prions to primate after an extended silent incubation period. Sci. Rep..

[25] van Duijn C.M., Delasnerie-Laupretre N., Masullo C., Zerr I., de Silva R., Wientjens D.P. (1998). Case-control study of risk factors of creutzfeldt-jakob disease in Europe during 1993-95. European union (EU) collaborative study group of creutzfeldt-jakob disease (CJD). Lancet.

[26] Cocco P.L., Caperna A., Vinci F. (2003). Occupational risk factors for the sporadic form of Creutzfeldt-Jakob disease. Med. Lav..

[27] Will R.G., Ironside J.W., Zeidler M., Cousens S.N., Estibeiro K., Alperovitch A. (1996). A new variant of Creutzfeldt-Jakob disease in the UK. Lancet.

[28] Wells G.A., Scott A.C., Johnson C.T., Gunning R.F., Hancock R.D., Jeffrey M. (1987). A novel progressive spongiform encephalopathy in cattle. Vet. Rec..

[29] Bruce M.E., Will R.G., Ironside J.W., McConnell I., Drummond D., Suttie A. (1997). Transmissions to mice indicate that 'new variant' CJD is caused by the BSE agent. Nature.

[30] Hill A.F., Desbruslais M., Joiner S., Sidle K.C., Gowland I., Collinge J. (1997). The same prion strain causes vCJD and BSE. Nature.

[31] Babelhadj B., Di Bari M.A., Pirisinu L., Chiappini B., Gaouar S.B.S., Riccardi G. (2018). Prion disease in dromedary camels, Algeria. Emerg. Infect. Dis..

[32] Benestad S.L., Mitchell G., Simmons M., Ytrehus B., Vikoren T. (2016). First case of chronic wasting disease in Europe in a Norwegian free-ranging reindeer. Vet. Res..

[33] Tranulis M.A., Gavier-Widen D., Vage J., Noremark M., Korpenfelt S.L., Hautaniemi M. (2021). Chronic wasting disease in Europe: new strains on the horizon. Acta Vet. Scand..

[34] Tranulis M.A., Tryland M. (2023). The zoonotic potential of chronic wasting disease-A review. Foods.

[35] Williams E.S., Young S. (1980). Chronic wasting disease of captive mule deer: a spongiform encephalopathy. J. Wildl. Dis..

[36] Mathiason C.K. (2023). Large animal models for chronic wasting disease. Cell Tissue Res..

[37] Kim T.Y., Shon H.J., Joo Y.S., Mun U.K., Kang K.S., Lee Y.S. (2005). Additional cases of chronic wasting disease in imported deer in Korea. J. Vet. Med. Sci..

[38] Hamir A.N., Kunkle R.A., Cutlip R.C., Miller J.M., O'Rourke K.I., Williams E.S. (2005). Experimental transmission of chronic wasting disease agent from mule deer to cattle by the intracerebral route. J. Vet. Diagn. Invest..

[39] Hamir A.N., Kunkle R.A., Cutlip R.C., Miller J.M., Williams E.S., Richt J.A. (2006). Transmission of chronic wasting disease of mule deer to Suffolk sheep following intracerebral inoculation. J. Vet. Diagn. Invest..

[40] Moore S.J., West Greenlee M.H., Kondru N., Manne S., Smith J.D., Kunkle R.A. (2017). Experimental transmission of the chronic wasting disease agent to swine after oral or intracranial inoculation. J. Virol..

[41] Cortez L.M., Morrison A.J., Garen C.R., Patterson S., Uyesugi T., Petrosyan R. (2022). Probing the origin of prion protein misfolding via reconstruction of ancestral proteins. Protein Sci..

[42] Thackray A.M., Andreoletti O., Bujdoso R. (2018). Mammalian prion propagation in PrP transgenic Drosophila. Brain.

[43] Thackray A.M., Andreoletti O., Spiropoulos J., Bujdoso R. (2021). A new model for sensitive detection of zoonotic prions by PrP transgenic Drosophila. J. Biol. Chem..

[44] Thackray A.M., McNulty E.E., Nalls A.V., Cardova A., Tran L., Telling G. (2023). Genetic modulation of CWD prion propagation in cervid PrP Drosophila. Biochem. J..

[45] Schatzl H.M., Da Costa M., Taylor L., Cohen F.E., Prusiner S.B. (1995). Prion protein gene variation among primates. J. Mol. Biol..

[46] Bischof J., Maeda R.K., Hediger M., Karch F., Basler K. (2007). An optimized transgenesis system for Drosophila using germ-line-specific phiC31 integrases. Proc. Natl. Acad. Sci. U. S. A..

[47] Thackray A.M., Di Y., Zhang C., Wolf H., Pradl L., Vorberg I. (2014). Prion-induced and spontaneous formation of transmissible toxicity in PrP transgenic Drosophila. Biochem. J..

[48] Raeber A.J., Muramoto T., Kornberg T.B., Prusiner S.B. (1995). Expression and targeting of Syrian hamster prion protein induced by heat shock in transgenic Drosophila melanogaster. Mech. Dev..

[49] Gavin B.A., Dolph M.J., Deleault N.R., Geoghegan J.C., Khurana V., Feany M.B. (2006). Accelerated accumulation of misfolded prion protein and spongiform degeneration in a Drosophila model of Gerstmann-Straussler-Scheinker syndrome. J. Neurosci..

[50] Bueler H., Aguzzi A., Sailer A., Greiner R.A., Autenried P., Aguet M. (1993). Mice devoid of PrP are resistant to scrapie. Cell.

[51] Mallucci G., Dickinson A., Linehan J., Klohn P.C., Brandner S., Collinge J. (2003). Depleting neuronal PrP in prion infection prevents disease and reverses spongiosis. Science.

[52] Fernandez-Borges N., Espinosa J.C., Marin-Moreno A., Aguilar-Calvo P., Asante E.A., Kitamoto T. (2017). Protective effect of Val(129)-PrP against bovine spongiform encephalopathy but not variant Creutzfeldt-Jakob disease. Emerg. Infect. Dis..

[53] Takeuchi A., Kobayashi A., Ironside J.W., Mohri S., Kitamoto T. (2013). Characterization of variant Creutzfeldt-Jakob disease prions in prion protein-humanized mice carrying distinct codon 129 genotypes. J. Biol. Chem..

[54] Mead S., Poulter M., Uphill J., Beck J., Whitfield J., Webb T.E. (2009). Genetic risk factors for variant Creutzfeldt-Jakob disease: a genome-wide association study. Lancet Neurol..

[55] Lasmezas C.I., Deslys J.P., Demaimay R., Adjou K.T., Lamoury F., Dormont D. (1996). BSE transmission to macaques. Nature.

[56] Herzog C., Sales N., Etchegaray N., Charbonnier A., Freire S., Dormont D. (2004). Tissue distribution of bovine spongiform encephalopathy agent in primates after intravenous or oral infection. Lancet.

[57] Wadsworth J.D., Asante E.A., Desbruslais M., Linehan J.M., Joiner S., Gowland I. (2004). Human prion protein with valine 129 prevents expression of variant CJD phenotype. Science.

[58] Mawhinney S., Pape W.J., Forster J.E., Anderson C.A., Bosque P., Miller M.W. (2006). Human prion disease and relative risk associated with chronic wasting disease. Emerg. Infect. Dis..

[59] Anderson C.A., Bosque P., Filley C.M., Arciniegas D.B., Kleinschmidt-Demasters B.K., Pape W.J. (2007). Colorado surveillance program for chronic wasting disease transmission to humans: lessons from 2 highly suspicious but negative cases. Arch. Neurol..

[60] Belay E.D., Gambetti P., Schonberger L.B., Parchi P., Lyon D.R., Capellari S. (2001). Creutzfeldt-Jakob disease in unusually young patients who consumed venison. Arch. Neurol..

[61] Olszowy K.M., Lavelle J., Rachfal K., Hempstead S., Drouin K., Darcy J.M. (2014). Six-year follow-up of a point-source exposure to CWD contaminated venison in an Upstate New York community: risk behaviours and health outcomes 2005-2011. Public Health.

[62] Hayasaka K., Gojobori T., Horai S. (1988). Molecular phylogeny and evolution of primate mitochondrial DNA. Mol. Biol. Evol..

[63] Hannaoui S., Cheng G., Wemheuer W., Schultz-Schaeffer W.J., Gilch S., Schatzl H.M. (2022). Prion 2022 Conference abstract (pp153): transmission of prion infectivity from CWD-infected macaque tissues to rodent models demonstrates the zoonotic potential of chronic wasting disease. Prion.

[64] Race B., Williams K., Orru C.D., Hughson A.G., Lubke L., Chesebro B. (2018). Lack of transmission of chronic wasting disease to cynomolgus macaques. J. Virol..

[65] Hannaoui S., Zemlyankina I., Chang S.C., Arifin M.I., Beringue V., McKenzie D. (2022). Transmission of cervid prions to humanized mice demonstrates the zoonotic potential of CWD. Acta Neuropathol..

[66] Ritchie D.L., Barria M.A. (2021). Prion diseases: a unique transmissible agent or a model for neurodegenerative diseases?. Biomolecules.

[67] Watts J.C., Giles K., Patel S., Oehler A., DeArmond S.J., Prusiner S.B. (2014). Evidence that bank vole PrP is a universal acceptor for prions. PLoS Pathog..

[68] Green R., Horrocks C., Wilkinson A., Hawkins S.A., Ryder S.J. (2005). Primary isolation of the bovine spongiform encephalopathy agent in mice: agent definition based on a review of 150 transmissions. J. Comp. Pathol..

[69] Bruce M., Chree A., McConnell I., Foster J., Pearson G., Fraser H. (1994). Transmission of bovine spongiform encephalopathy and scrapie to mice: strain variation and the species barrier. Philos. Trans. R. Soc. Lond. B Biol. Sci..

[70] Ghosh S. (2018). Sialylation and sialyltransferase in insects. Glycoconj J..

[71] Werz D.B., Ranzinger R., Herget S., Adibekian A., von der Lieth C.W., Seeberger P.H. (2007). Exploring the structural diversity of mammalian carbohydrates ("glycospace") by statistical databank analysis. ACS Chem. Biol..

[72] Katorcha E., Makarava N., Savtchenko R., D'Azzo A., Baskakov I.V. (2014). Sialylation of prion protein controls the rate of prion amplification, the cross-species barrier, the ratio of PrPSc glycoform and prion infectivity. PLoS Pathog..

[73] Thackray A.M., Lam B., McNulty E.E., Nalls A.V., Mathiason C.K., Magadi S.S. (2022). Clearance of variant Creutzfeldt-Jakob disease prions in vivo by the Hsp70 disaggregase system. Brain.

[74] Supattapone S. (2020). Cofactor molecules: essential partners for infectious prions. Prog. Mol. Biol. Transl Sci..

[75] Myers R., Cembran A., Fernandez-Funez P. (2020). Insight from animals resistant to prion diseases: deciphering the genotype - morphotype - phenotype code for the prion protein. Front Cell Neurosci..

[76] Green C., Levashina E., McKimmie C., Dafforn T., Reichhart J.M., Gubb D. (2000). The necrotic gene in Drosophila corresponds to one of a cluster of three serpin transcripts mapping at 43A1.2. Genetics.

[77] Padilla D., Beringue V., Espinosa J.C., Andreoletti O., Jaumain E., Reine F. (2011). Sheep and goat BSE propagate more efficiently than cattle BSE in human PrP transgenic mice. PLoS Pathog..

[78] Notari S., Xiao X., Espinosa J.C., Cohen Y., Qing L., Aguilar-Calvo P. (2014). Transmission characteristics of variably protease-sensitive prionopathy. Emerg. Infect. Dis..

[79] Thackray A.M., Hopkins L., Spiropoulos J., Bujdoso R. (2008). Molecular and transmission characteristics of primary-passaged ovine scrapie isolates in conventional and ovine PrP transgenic mice. J. Virol..

[80] Feraudet C., Morel N., Simon S., Volland H., Frobert Y., Creminon C. (2005). Screening of 145 anti-PrP monoclonal antibodies for their capacity to inhibit PrPSc replication in infected cells. J. Biol. Chem..

[81] McNulty E., Nalls A.V., Mellentine S., Hughes E., Pulscher L., Hoover E.A. (2019). Comparison of conventional, amplification and bio-assay detection methods for a chronic wasting disease inoculum pool. PLoS One.

[82] Nalls A.V., McNulty E., Hoover C.E., Pulscher L.A., Hoover E.A., Mathiason C.K. (2017). Infectious prions in the pregnancy microenvironment of Chronic Wasting Disease-infected Reeves' muntjac deer. J. Virol..

[83] Henderson D.M., Manca M., Haley N.J., Denkers N.D., Nalls A.V., Mathiason C.K. (2013). Rapid antemortem detection of CWD prions in deer saliva. PLoS One.

[84] Huor A., Douet J.Y., Lacroux C., Lugan S., Tillier C., Aron N. (2017). Infectivity in bone marrow from sporadic CJD patients. J. Pathol..

[85] Castilla J., Gutierrez Adan A., Brun A., Pintado B., Ramirez M.A., Parra B. (2003). Early detection of PrPres in BSE-infected bovine PrP transgenic mice. Arch. Virol..

[86] Fraser H., Dickinson A.G. (1968). The sequential development of the brain lesion of scrapie in three strains of mice. J. Comp. Pathol..

