# Supplementary material for: Lack of prion transmission barrier in human PrP transgenic Drosophila
Source: J Biol Chem. 2024 Jul 30;300(9):107617. doi: 10.1016/j.jbc.2024.107617 (PMC11386037; doi:10.1016/j.jbc.2024.107617)
Supplement: Supporting Information Tables and Figures [file mmc1.pdf]

Title: Lack of prion transmission barrier in human PrP transgenic *Drosophila*

Authors: Alana M. Thackray, Erin E. McNulty, Amy V. Nalls, Andrew Smith, Emmanuel Comoy Glenn Telling, Sylvie L. Benestad, Olivier Andréoletti, Candace K. Mathiason and Raymond Bujdoso

List of material included:

Supporting Information Table S1
Supporting Information Figure S1
Supporting Information Table S2
Supporting Information Figure S2
Supporting Information Table S3
Supporting Information Table S4
Supporting Information Table S5
Supporting Information Table S6
Supporting Information Table S7
Supporting Information Figure S3
Supporting Information Table S8
Supporting Information Table S9A
Supporting Information Table S9B

Supporting Information Table S1. Statistical analysis of prion seeding activity in prion-exposed primate PrP *Drosophila*

Human vCJD

Fly line	Median	*p*-value	Significance
**Control 51D**			
Day 5	0	>0.9999	NS
Day 10	0	>0.9999	NS
Day 20	0	>0.9999	NS
Day 30	0	>0.9999	NS
Day 40	0	0.2500	NS
**M129 PrP**			
Day 5	0	>0.9999	NS
Day 10	0	>0.9999	NS
Day 20	0.0021	0.3506	NS
Day 30	0.0281	0.0156	*
Day 40	0.1546	0.0002	***
**V129 PrP**			
Day 5	0	>0.9999	NS
Day 10	0	>0.9999	NS
Day 20	0	>0.9999	NS
Day 30	0	0.2000	NS
Day 40	0	>0.9999	NS
**Chimpanzee PrP**			
Day 5	0	>0.9999	NS
Day 10	0	>0.9999	NS
Day 20	0.0554	0.0014	**
Day 30	0.0632	0.0078	**
Day 40	0.1534	0.0002	***
**Macaque PrP**			
Day 5	0	>0.9999	NS
Day 10	0	>0.9999	NS
Day 20	0	>0.9999	NS
Day 30	0.0242	0.0156	*
Day 40	0.0667	0.0002	***
**Squirrel monkey PrP**			
Day 5	0	>0.9999	NS
Day 10	0	>0.9999	NS
Day 20	0	>0.9999	NS
Day 30	0.0385	0.0078	**
Day 40	0.0357	0.0002	***
**Lemur PrP**			
Day 5	0	>0.9999	NS
Day 10	0	>0.9999	NS
Day 20	0	>0.9999	NS
Day 30	0	>0.9999	NS
Day 40	0.0289	0.0156	*

Bovine classical BSE

Fly line	Median	*p*-value	Significance
**Control 51D**			
Day 5	0	>0.9999	NS
Day 10	0	0.5000	NS
Day 20	0	>0.9999	NS
Day 30	0	0.3538	NS
Day 40	0	>0.9999	NS
**M129 PrP**			
Day 5	0	>0.9999	NS
Day 10	0	0.4667	NS
Day 20	0	0.7333	NS
Day 30	0.5284	0.0078	**
Day 40	0.0491	0.0002	***
**V129 PrP**			
Day 5	0	>0.9999	NS
Day 10	0	>0.9999	NS
Day 20	0	>0.9999	NS
Day 30	0.0504	0.0078	**
Day 40	0.0688	0.0002	***
**Chimpanzee PrP**			
Day 5	0	>0.9999	NS
Day 10	0	>0.9999	NS
Day 20	0	>0.9999	NS
Day 30	0	>0.9999	NS
Day 40	0.5032	0.0078	**
**Macaque PrP**			
Day 5	0	>0.9999	NS
Day 10	0.0087	0.0769	NS
Day 20	0	>0.9999	NS
Day 30	0	0.5000	NS
Day 40	0.0896	0.0078	**
**Squirrel monkey PrP**			
Day 5	0	>0.9999	NS
Day 10	0	0.7333	NS
Day 20	0	>0.9999	NS
Day 30	0	>0.9999	NS
Day 40	0.0628	0.0002	***
**Lemur PrP**			
Day 5	0	>0.9999	NS
Day 10	0	0.4667	NS
Day 20	0	>0.9999	NS
Day 30	0	0.5000	NS
Day 40	0.0681	0.0312	*

Cervid CWD

Fly line	Median	*p*-value	Significance
**Control 51D**			
Day 5	0	>0.9999	NS
Day 10	0	>0.9999	NS
Day 20	0	>0.9999	NS
Day 30	0	>0.9999	NS
Day 40	0	>0.9999	NS
**M129 PrP**			
Day 5	0	>0.9999	NS
Day 10	0	0.2500	NS
Day 20	0	>0.9999	NS
Day 30	0.4053	0.0002	***
Day 40	0.5473	0.0078	**
**V129 PrP**			
Day 5	0	>0.9999	NS
Day 10	0	>0.9999	NS
Day 20	0.4602	0.0002	***
Day 30	0.5492	0.0078	**
Day 40	0.6647	0.0002	***
**Chimpanzee PrP**			
Day 5	0	>0.9999	NS
Day 10	0	>0.9999	NS
Day 20	0	>0.9999	NS
Day 30	0.3163	0.0078	**
Day 40	0.4484	0.0078	**
**Macaque PrP**			
Day 5	0	>0.9999	NS
Day 10	0	0.5000	NS
Day 20	0	>0.9999	NS
Day 30	0.3033	0.0078	**
Day 40	0.5724	0.0078	**
**Squirrel monkey PrP**			
Day 5	0	>0.9999	NS
Day 10	0	>0.9999	NS
Day 20	0.4695	0.0078	**
Day 30	0.4880	0.0002	***
Day 40	0.4217	0.0078	**
**Lemur PrP**			
Day 5	0	>0.9999	NS
Day 10	0	>0.9999	NS
Day 20	0	>0.9999	NS
Day 30	0.4960	0.0078	**
Day 40	0.4090	0.0002	***

Supporting Information Table S1. Statistical analysis of prion seeding activity in prion-exposed primate PrP *Drosophila*

Elav x primate PrP *Drosophila* and *Elav* x control 51D *Drosophila* were exposed to human vCJD, bovine classical BSE, or cervid CWD (white-tailed deer CWD prion-infected), or prion-free human, bovine, or cervid normal brain homogenate at the larval stage. Adult *Drosophila* were collected at the indicated time points after hatching (day 5, 10, 20, 30 or 40), and head homogenate was prepared and used as seed in RT-QuIC reactions. Statistical analysis of the prion seeding activity in Figure 3 was assessed by the Mann-Whitney or Wilcoxon test to generate *p*-values (those <0.05 were considered significant) by comparing the median of the prion-exposed sample rates to the median of the control treatment rates. NS = not significant.

Supporting Information Figure S1. Lack of accelerated decline of locomotor activity in prion-exposed control 51D *Drosophila*

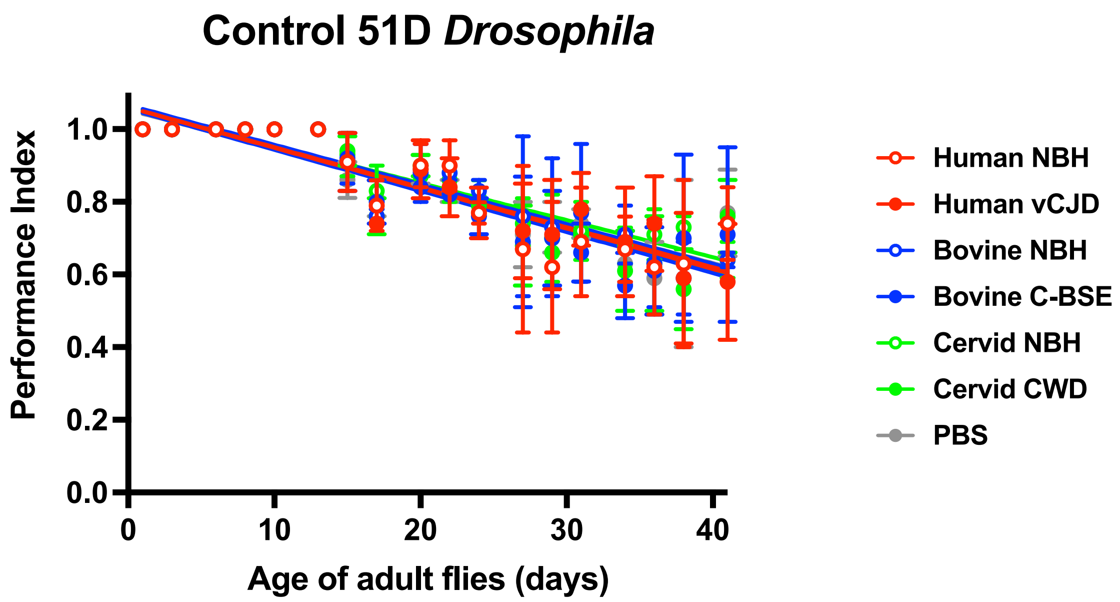

Supporting Information Figure S1. Lack of accelerated decline of locomotor activity in prion-exposed control 51D *Drosophila*
Elav x control 51D *Drosophila* were exposed to human vCJD, bovine classical BSE or white-tailed deer CWD prion-infected, or control normal (prion-free) human, bovine or cervid brain homogenate, respectively, at the larval stage. After hatching, flies were assessed for locomotor activity by a negative geotaxis climbing assay. The mean performance index was determined for three groups of n=15 flies of each genotype per time point. Statistical analysis of Figure 4 and Supporting Information Figure S1 is shown in Supporting Information Table S2. NBH = normal brain homogenate; vCJD = variant CJD; C-BSE = classical BSE; CWD = chronic wasting disease.

Supporting Information Table S2. Statistical analysis of accelerated decline of locomotor activity in prion-exposed primate PrP *Drosophila*

Drosophila Fly line	Inoculum comparison: vCJD vs Human NBH	C-BSE vs Bovine NBH	WTD-CWD vs Cervid NBH
**M129 human PrP**	***p*=0.0087**	***p*=0.0062**	***p*=0.0146**
**V129 human PrP**	***p*=0.2645**	***P*=0.5136**	***p*=0.0024**
**Chimpanzee PrP**	***p*=0.0082**	***p*=0.0014**	***p*=0.0058**
**Cynomolgus macaque PrP**	***p*=0.0077**	***p*=0.0010**	***p*=0.0030**
**Squirrel monkey PrP**	***p*=0.0110**	***p*=0.0031**	***p*<0.0001**
**Lemur PrP**	***p*<0.0001**	***p*=0.0040**	***p*=0.0054**
**Control 51D**	***p*=0.9563**	***P*=0.7141**	***P*=0.7185**

Supporting Information Table S2. Statistical analysis of accelerated decline of locomotor activity in prion-exposed primate PrP *Drosophila*
Elav x primate PrP and *Elav* x control 51D *Drosophila* were exposed to human vCJD, bovine classical BSE or white-tailed deer CWD prion-infected, or control normal (prion-free) human, bovine or cervid brain homogenate, respectively, at the larval stage. After hatching, flies were assessed for locomotor activity by a negative geotaxis climbing assay. The mean performance index was determined for three groups of n=15 flies of each genotype per time point (see Figure 4 and Supporting Information Figure S1). Statistical analysis was performed using an unpaired (two-tailed) Student t test with each prion treatment group compared to their respective prion-free control group over the whole of the climbing assay time course. Where $p<0.05$ comparisons are statistically significantly different.
vCJD = variant CJD; C-BSE= classical BSE; WTD = white-tailed deer; CWD = chronic wasting disease; NBH = normal brain homogenate.

Supporting Information Figure S2. Lack of accelerated loss of survival of prion-exposed control 51D *Drosophila*

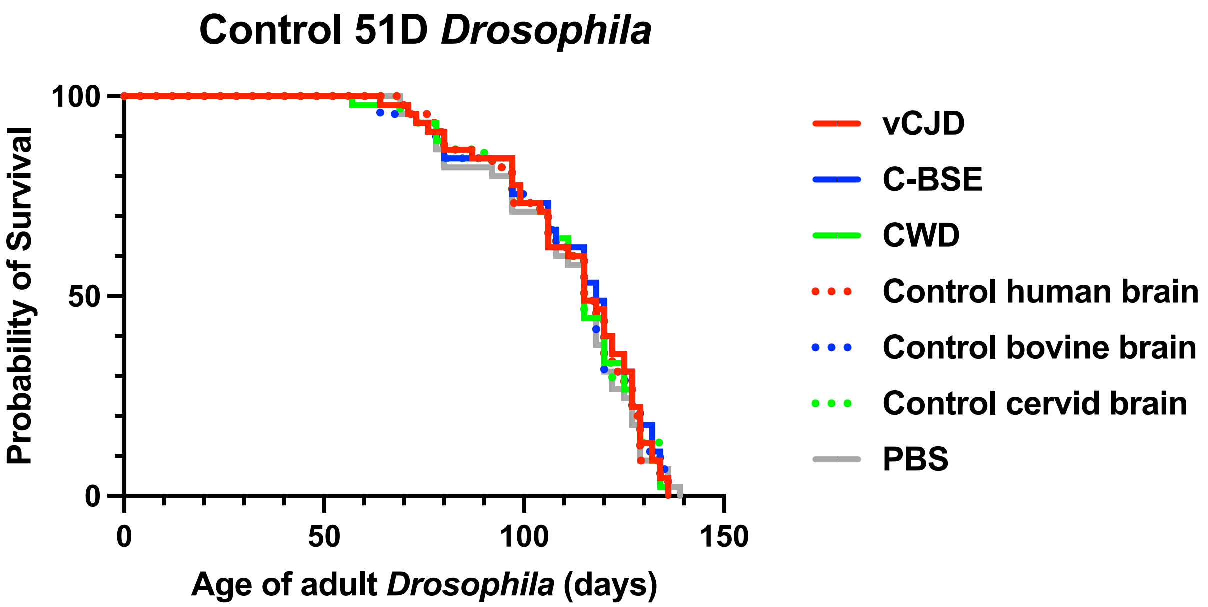

Supporting Information Figure S2. Lack of accelerated loss of survival of prion-exposed control 51D *Drosophila*
Elav x control 51D *Drosophila* were exposed to human vCJD, bovine classical BSE or white-tailed deer CWD prion-infected, or control normal (prion-free) human, bovine or cervid brain homogenate, respectively, or PBS, at the larval stage. After hatching, the number of surviving flies was recorded three times a week and the data displayed as Kaplan-Meier plots (see Figure 5). For statistical analysis of accelerated loss of survival of prion-exposed primate PrP *Drosophila*, see Supporting Information Table S3. vCJD = variant CJD; C-BSE = classical BSE; CWD = chronic wasting disease.

Supporting Information Table S3. Statistical analysis of accelerated loss of survival of prion-exposed primate PrP *Drosophila*

	Median survival time in days						
***Drosophila* Fly line**	**vCJD**	**Human NBH**	**C-BSE**	**Bovine NBH**	**WTD-CWD**	**Cervid NBH**	**PBS**
**M129 human PrP**	**69***	**115**	**71***	**118**	**69***	**118**	**115**
**V129 human PrP**	**73***	**115**	**73***	**115**	**62***	**115**	**115**
**Chimpanzee PrP**	**78***	**115**	**64***	**115**	**85***	**115**	**115**
**Cynomolgus macaque PrP**	**62***	**115**	**66***	**115**	**69***	**115**	**118**
**Squirrel monkey PrP**	**69***	**115**	**97***	**115**	**80***	**115**	**115**
**Lemur PrP**	**73***	**115**	**76***	**115**	**76***	**115**	**115**
**Control 51D**	**115**	**115**	**118**	**115**	**115**	**115**	**115**

Supporting Information Table S3. Statistical analysis of accelerated loss of survival of prion-exposed primate PrP *Drosophila*
Elav x primate PrP and *Elav* x control 51D *Drosophila* were exposed to human vCJD, bovine classical BSE or white-tailed deer CWD prion-infected, or control normal (prion-free) human, bovine or cervid brain homogenate, respectively, or PBS, at the larval stage. After hatching, the number of surviving flies was recorded three times a week and the data displayed as Kaplan-Meier plots (see Figure 5 and Supporting Information Figure S2) from which median survival times were determined. Statistical analysis of survival curve comparison (control prion-free treatment group versus prion treatment group for each fly line) was performed by the Log-rank (Mantel-Cox) test.
***** = $p<0.0001$ for each prion-infected group in comparison with its relevant NBH control treated group.
vCJD = variant CJD; C-BSE= classical BSE; WTD = white-tailed deer; NBH = normal brain homogenate.

Supporting Information Table S4. Statistical analysis of prion seeding activity in CWD prion-exposed human PrP *Drosophila*

M129 human PrP *Drosophila*

CWD inoculum	Median	*p*-value	Significance
**White-tailed deer brain**			
Day 5	0	0.0769	NS
Day 10	0	>0.9999	NS
Day 20	0.2625	0.0078	**
Day 30	0.3936	0.0002	***
Day 40	0.3373	0.0078	**
**Muntjac brain**			
Day 5	0	0.1795	NS
Day 10	0	>0.9999	NS
Day 20	0	>0.9999	NS
Day 30	0.2632	0.0002	***
Day 40	0.3087	0.0078	**
**Reindeer brain**			
Day 5	0	0.0769	NS
Day 10	0	>0.9999	NS
Day 20	0	>0.9999	NS
Day 30	0.1359	0.0005	***
Day 40	0.0546	0.0156	*
**Reindeer lymph node**			
Day 5	0	0.7063	NS
Day 10	0	>0.9999	NS
Day 20	0.1415	0.0078	**
Day 30	0.1122	0.0002	***
Day 40	0.1606	0.0078	**
**Moose brain**			
Day 5	0	0.0769	NS
Day 10	0	>0.9999	NS
Day 20	0.3027	0.0078	**
Day 30	0.2579	0.0002	***
Day 40	0.4347	0.0078	**

V129 human PrP *Drosophila*

CWD inoculum	Median	*p*-value	Significance
**White-tailed deer brain**			
Day 5	0	>0.9999	NS
Day 10	0	>0.9999	NS
Day 20	0.2158	0.0002	***
Day 30	0.3365	0.0002	***
Day 40	0.2825	0.0002	***
**Muntjac brain**			
Day 5	0	>0.9999	NS
Day 10	0	>0.9999	NS
Day 20	0	>0.9999	NS
Day 30	0.3325	0.0002	***
Day 40	0.2514	0.0002	***
**Reindeer brain**			
Day 5	0	>0.9999	NS
Day 10	0	>0.9999	NS
Day 20	0	>0.9999	NS
Day 30	0	>0.9999	NS
Day 40	0.0346	0.2124	NS
**Reindeer lymph node**			
Day 5	0	0.4667	NS
Day 10	0	>0.9999	NS
Day 20	0	>0.9999	NS
Day 30	0.0918	0.0002	***
Day 40	0.1266	0.0044	**
**Moose brain**			
Day 5	0	0.4667	NS
Day 10	0	>0.9999	NS
Day 20	0	>0.9999	NS
Day 30	0.2326	0.0002	***
Day 40	0.0276	0.0002	***

Control 51D *Drosophila*

CWD inoculum	Median	*p*-value	Significance
**White-tailed deer brain**			
Day 5	0	>0.9999	NS
Day 10	0	>0.9999	NS
Day 20	0	>0.9999	NS
Day 30	0	>0.9999	NS
Day 40	0	>0.9999	NS
**Muntjac brain**			
Day 5	0	>0.9999	NS
Day 10	0	>0.9999	NS
Day 20	0	>0.9999	NS
Day 30	0	>0.9999	NS
Day 40	0	>0.9999	NS
**Reindeer brain**			
Day 5	0	>0.9999	NS
Day 10	0	>0.9999	NS
Day 20	0	>0.9999	NS
Day 30	0	0.5000	NS
Day 40	0	>0.9999	NS
**Reindeer lymph node**			
Day 5	0	>0.9999	NS
Day 10	0	>0.9999	NS
Day 20	0	>0.9999	NS
Day 30	0	>0.9999	NS
Day 40	0	>0.9999	NS
**Moose brain**			
Day 5	0	>0.9999	NS
Day 10	0	>0.9999	NS
Day 20	0	>0.9999	NS
Day 30	0	>0.9999	NS
Day 40	0	>0.9999	NS

Supporting Information Table S4. Statistical analysis of prion seeding activity in CWD prion-exposed human PrP *Drosophila*
Elav x M129 and *Elav* x V129 human PrP *Drosophila* and *Elav* x control 51D *Drosophila* were exposed to CWD-infected North American (white-tailed deer or muntjac) or European (Norwegian reindeer or moose) brain material, or Norwegian reindeer lymph node material or prion-free control cervid normal brain homogenate (NBH in Figure 7) at the larval stage. Adult *Drosophila* were collected at the indicated time points after hatching, and head homogenate was prepared and used as seed in RT-QuIC reactions. Statistical analysis of the prion seeding activity in Figure 7 was assessed by the Mann-Whitney or Wilcoxon test to generate *p*-values (those <0.05 were considered significant) by comparing the median of the CWD-exposed sample rates to the median of the control treatment rates. NS = not significant.

Supporting Information Table S5. Statistical analysis of accelerated loss of locomotor activity in CWD prion-exposed human PrP *Drosophila*

	Inocula					
**Fly line**	**White-tailed deer**	**Muntjac**	**Reindeer**	**Reindeer LN**	**Moose**	**PBS**
**M129 human PrP**	***p*=0.0142**	***p*=0.0136**	***p*=0.0106**	***P*=0.0221**	***P*=0.0278**	***p*=0.0008**
**V129 human PrP**	***p*=0.0141**	***P*=0.0195**	***p*=0.0206**	***p*=0.0296**	***p*=0.0285**	***p*=0.0212**
**Control 51D**	***p*=0.6416**	***p*=0.8903**	***p*=0.6871**	***p*=0.9617**	***p*=0.3400**	***p*=0.8123**

Supporting Information Table S5. Statistical analysis of accelerated loss of locomotor activity in CWD prion-exposed human PrP *Drosophila*
Elav x M129 or *Elav* x V129 human PrP *Drosophila* and *Elav* x control 51D *Drosophila* were exposed to a 10^{-2} dilution of CWD-infected white-tailed deer, muntjac, reindeer or moose brain, or reindeer lymph node (LN), or normal (prion-free) cervid brain homogenate, or PBS, at the larval stage. After hatching, flies were assessed for locomotor activity by a negative geotaxis climbing assay. The mean performance index was determined for three groups of n=15 flies of each genotype per time point (see Figure 8). Statistical analysis was performed using an unpaired (two-tailed) Student t test with each prion treatment group compared to the prion-free control group over the whole of the climbing assay time course. Where $p<0.05$ comparisons are statistically significantly different.

Supporting Information Table S6. Statistical analysis of accelerated loss of survival of CWD prion-exposed human PrP *Drosophila*

Fly line	Median survival time in days (*p* value for Kaplan-Meier plot comparison)						
	**Control NBH**	**White-tailed deer**	**Muntjac**	**Reindeer**	**Reindeer LN**	**Moose**	**PBS**
**M129 human PrP**	**106**	**85 (p=0.0032)**	**85 (p=0.0011)**	**26 (p<0.0001)**	**78 (p=0.0141)**	**106 (p=0.9107)**	**106 (p=0.0018)**
**V129 human PrP**	**85**	**57 (p<0.0001)**	**40 (p<0.0001)**	**57 (p<0.0001)**	**33 (p<0.0001)**	**82 (p=0.3174)**	**78 (p=0.9238)**
**Control 51D**	**127**	**129 (p=0.0232)**	**134 (p=0.0571)**	**131 (p=0.7195)**	**138 (p=0.3801)**	**131 (p=0.0032)**	**122 (p=0.0165)**

Supporting Information Table S6. Statistical analysis of accelerated loss of survival of CWD prion-exposed human PrP *Drosophila*
Elav x M129 or *Elav* x V129 human PrP *Drosophila* and *Elav* x control 51D *Drosophila* were exposed to a 10^{-2} dilution of CWD-infected white-tailed deer, muntjac, reindeer or moose brain, or reindeer lymph node (LN), or normal (prion-free) cervid brain homogenate, or PBS, at the larval stage. After hatching, the number of surviving flies was recorded three times a week and the data displayed as Kaplan-Meier plots (see Figure 9) from which median survival times were determined. Statistical analysis of survival curve comparison (prion-free control treatment group versus each individual other treatment group for each fly line) was performed by the Log-rank (Mantel-Cox) test. Where p<0.05 comparisons are statistically significantly different.
NBH = normal cervid brain homogenate.

Supporting Information Table S7. Statistical analysis of accelerated decline of locomotor activity in prion-exposed *Drosophila*

	Drosophila fly line					
	Cervid PrP		V129 human PrP		Control 51D	
Dilution	Reindeer	Moose	Reindeer	Moose	Reindeer	Moose
10^{-2}	***p***<0.0001	***p***<0.0001	***p***<0.0001	***p***=0.0001	***p***=0.8846	***p***=0.5116
10^{-4}	***p***<0.0001	***p***=0.0004	***p***<0.0001	***p***=0.0001	***p***=0.6418	***p***=0.3984
10^{-6}	***p***=0.0002	***p***=0.0010	***p***=0.0001	***p***=0.0001	***p***=0.4419	***p***=0.1638
10^{-8}	***p***=0.0011	***p***=0.0046	***p***=0.0001	***p***=0.0005	***p***=0.3257	***p***=0.2898
10^{-10}	***p***=0.0208	***p***=0.0093	***p***=0.0001	***p***=0.0005	***p***=0.4970	***p***=0.5532
10^{-12}	***p***=0.0250	***p***=0.4601	***p***=0.0004	***p***=0.0030	***p***=0.4884	***p***=0.3216
10^{-14}	***p***=0.0894	***p***=0.1383	***p***=0.0009	***p***=0.0004	***p***=0.7108	***p***=0.7760
10^{-16}	***p***=0.2209	***p***=0.3526	***p***=0.0016	***p***=0.0067	***p***=0.5602	***p***=0.2192
10^{-18}	***p***=0.3831	***p***=0.7544	***p***=0.0033	***p***=0.0144	***p***=0.4549	***p***=0.1920
PBS	***p***=0.8540	***p***=0.9699	***p***=0.0005	***p***=0.1107	***p***=0.8807	***p***=0.6391

Supporting Information Table S7. Statistical analysis of accelerated decline of locomotor activity in prion-exposed *Drosophila*

Elav x cervid PrP, *Elav* x V129 human PrP, and *Elav x* control 51D *Drosophila* were exposed to a 10^{-2} dilution series of CWD-infected Norwegian reindeer or moose cervid brain homogenate or a 10^{-2} dilution of normal (prion-free) cervid brain homogenate, or PBS, at the larval stage. After hatching, flies were assessed for locomotor activity by a negative geotaxis climbing assay. The mean performance index was determined for three groups of n=15 flies of each genotype per time point (see Figure 10). Statistical analysis was performed using an unpaired (two-tailed) Student t test with each prion treatment group compared to the prion-free control cervid brain homogenate treatment group over the whole of the climbing assay time course. Where $p<0.05$ comparisons are statistically significantly different.

Supporting Information Figure S3. Accelerated loss of locomotor activity induced by reindeer or moose CWD prions

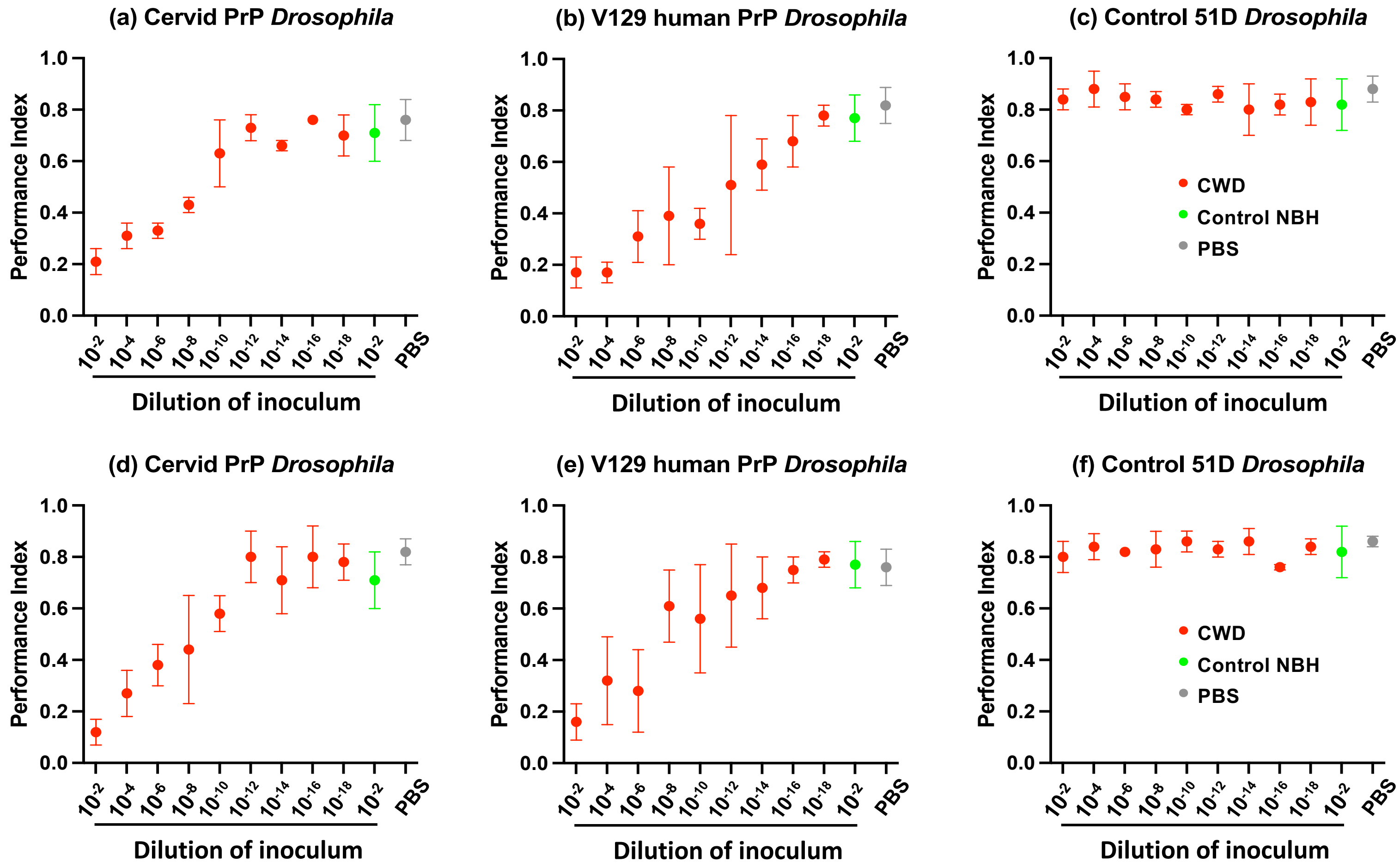

Supporting Information Figure S3. Accelerated loss of locomotor activity induced by reindeer or moose CWD prions

Elav x cervid PrP (a) and (d), *Elav* x V129 human PrP (b) and (e) or *Elav* x control 51D (c) and (f) *Drosophila* were exposed to a 10^{-2} dilution series of European (Norwegian reindeer or moose) CWD-infected brain material, or a 10^{-2} dilution of prion-free control cervid normal brain homogenate (control NBH), or PBS, at the larval stage. After hatching, flies were assessed for locomotor activity by a negative geotaxis climbing assay. The mean performance index was determined for three groups of n=15 flies of each genotype per time point (see Figure 10). Statistical analysis was performed using an unpaired (two-tailed) Student t test with each prion treatment group compared to the prion-free control cervid brain homogenate treatment group over the whole of the climbing assay time course (see Supporting Information Table S7). Day 50 mean performance index data are shown here (±SD). Reindeer CWD prions (a) - (c) and moose CWD prions (d) - (f).

Supporting Information Table S8. Statistical analysis of prion seeding activity in prion-exposed cervid or human PrP *Drosophila*

Cervid PrP *Drosophila*

CWD inoculum	Median	*p*-value	Significance
**Reindeer brain**			
10-2	0.1439	0.0078	**
10-4	0.1076	0.0078	**
10-6	0.0862	0.0078	**
10-8	0.0658	0.0156	*
10-10	0.1315	0.0078	**
10-12	0	>0.9999	NS
10-14	0	>0.9999	NS
10-16	0	>0.9999	NS
10-18	0	0.5000	NS
**Moose brain**			
10-2	0.2586	0.0078	**
10-4	0.5063	0.0078	**
10-6	0.3733	0.0078	**
10-8	0.2901	0.0078	**
10-10	0	>0.9999	NS
10-12	0	>0.9999	NS
10-14	0	>0.9999	NS
10-16	0.0023	0.3506	NS
10-18	0	0.5000	NS

V129 human PrP *Drosophila*

CWD inoculum	Median	*p*-value	Significance
**Reindeer brain**			
10-2	0.0583	0.0078	**
10-4	0.0653	0.0078	**
10-6	0.0339	0.0312	*
10-8	0	0.5000	NS
10-10	0	>0.9999	NS
10-12	0	0.5000	NS
10-14	0	>0.9999	NS
10-16	0	>0.9999	NS
10-18	0	0.5000	NS
**Moose brain**			
10-2	0.3151	0.0078	**
10-4	0	0.5000	NS
10-6	0	>0.9999	NS
10-8	0	>0.9999	NS
10-10	0	>0.9999	NS
10-12	0	>0.9999	NS
10-14	0	>0.9999	NS
10-16	0	>0.9999	NS
10-18	0	>0.9999	NS

Supporting Information Table S8. Statistical analysis of prion seeding activity accumulation induced by Norwegian reindeer or moose CWD inoculum

Elav x cervid PrP or *Elav* x V129 human PrP *Drosophila* were exposed to a 10^{-2} dilution series of European (Norwegian reindeer or moose) CWD-infected brain material or 10^{-2} prion-free control cervid normal brain homogenate at the larval stage. Adult *Drosophila* were collected at 40 days post hatching and head homogenate was prepared and used as seed in RT-QuIC reactions (see Figure 11). Statistical analysis of the prion seeding activity was assessed by the Mann-Whitney or Wilcoxon test to generate *p*-values (those <0.05 were considered significant) by comparing the median of the CWD-exposed sample rates to the median of the control treatment rates. NS = not significant.

Supporting Information Table S9A. Statistical analysis of accelerated decline of survival in prion-exposed *Drosophila*

Fly line	Control NBH	Median survival time in days (*p*-value) — Dilution of Reindeer CWD inoculum								
		10^{-2}	10^{-4}	10^{-6}	10^{-8}	10^{-10}	10^{-12}	10^{-14}	10^{-16}	10^{-18}
**Cervid PrP**	**122**	**76 (p<0.0001)**	**71 (p<0.0001)**	**85 (p<0.0001)**	**87 (p<0.0001)**	**78 (p<0.0001)**	**90 (p<0.0001)**	**92 (p<0.0001)**	**90 (p<0.0001)**	**120 (p=0.7123)**
**V129 human PrP**	**108**	**64 (p<0.0001)**	**76 (p<0.0001)**	**85 (p<0.0001)**	**87 (p<0.0001)**	**87 (p<0.0001)**	**83 (p<0.0001)**	**87 (p=0.0003)**	**90 (p=0.4259)**	**99 (p=0.3785)**
**Control 51D**	**118**	**115 (p=0.6254)**	**118 (p=0.5762)**	**115 (p=0.6228)**	**115 (p=0.6924)**	**118 (p=0.6423)**	**118 (p=0.5269)**	**115 (p=0.6816)**	**120 (p=0.5372)**	**118 (p=0.5393)**

Supporting Information Table S9A. Statistical analysis of accelerated decline of survival in prion-exposed *Drosophila*
Elav x cervid PrP and *Elav* x V129 human PrP *Drosophila,* and *Elav x* control 51D *Drosophila* were exposed to a 10^{-2} dilution series of CWD-infected Norwegian reindeer cervid brain material, or a 10^{-2} dilution of normal (prion-free) cervid brain homogenate, at the larval stage. After hatching, the number of surviving flies was recorded three times a week and the data displayed as Kaplan-Meier plots (see Figure 12a to 12c) from which median survival times were determined. Statistical analysis of survival curve comparison (prion-free control treatment group versus each individual other treatment group for each fly line) was performed by the Log-rank (Mantel-Cox) test. Where p<0.05 comparisons are statistically significantly different. NBH = normal cervid brain homogenate.

Supporting Information Table S9B. Statistical analysis of accelerated decline of survival in prion-exposed *Drosophila*

	Median survival time in days (*p*-value)									
		Dilution of Moose CWD inoculum								
**Fly line**	**Control NBH**	**10^{-2}**	**10^{-4}**	**10^{-6}**	**10^{-8}**	**10^{-10}**	**10^{-12}**	**10^{-14}**	**10^{-16}**	**10^{-18}**
**Cervid PrP**	**122**	**76 (p<0.0001)**	**76 (p<0.0001)**	**85 (p<0.0001)**	**87 (p<0.0001)**	**97 (p<0.0001)**	**97 (p<0.0001)**	**104 (p=0.0648)**	**120 (p=0.9143)**	**120 (p=0.7818)**
**V129 human PrP**	**108**	**106 (p=0.1280)**	**106 (p=0.1453)**	**106 (p=0.1204)**	**106 (p=0.2200)**	**104 (p=0.1642)**	**108 (p=0.1360)**	**106 (p=0.1884)**	**106 (p=0.1641)**	**108 (p=0.2186)**
**Control 51D**	**118**	**120 (p=0.7353)**	**118 (p=0.1220)**	**120 (p=0.4168)**	**122 (p=0.0237)**	**120 (p=0.2707)**	**120 (p=0.8669)**	**115 (p=0.8840)**	**115 (p=0.9082)**	**120 (p=0.6415)**

Supporting Information Table S9B. Statistical analysis of accelerated decline of survival in prion-exposed *Drosophila*

Elav x cervid PrP and *Elav* x V129 human PrP *Drosophila,* and *Elav x* control 51D *Drosophila* were exposed to a 10^{-2} dilution series of CWD-infected Norwegian moose cervid brain material, or a 10^{-2} dilution of normal (prion-free) cervid brain homogenate, at the larval stage. After hatching, the number of surviving flies was recorded three times a week and the data displayed as Kaplan-Meier plots (see Figure 12d to 12f) from which median survival times were determined. Statistical analysis of survival curve comparison (prion-free control treatment group versus each individual other treatment group for each fly line) was performed by the Log-rank (Mantel-Cox) test. Where $p<0.05$ comparisons are statistically significantly different. NBH = normal cervid brain homogenate.
